# An electrostatics method for converting a time-series into a weighted complex network

**DOI:** 10.1038/s41598-021-89552-2

**Published:** 2021-06-03

**Authors:** Dimitrios Tsiotas, Lykourgos Magafas, Panos Argyrakis

**Affiliations:** 1 Department of Regional and Economic Development, Agricultural University of Athens, Amfissa, Greece; 2 Adjunct Academic Staff, School of Social Sciences, Hellenic Open University, 10677 Athens, Greece; 3 Laboratory of Complex Systems, Department of Physics, International Hellenic University, Kavala, Greece; 4 Department of Physics, Aristotle University of Thessaloniki, Thessaloniki, Greece

**Keywords:** Mathematics and computing, Physics

## Abstract

This paper proposes a new method for converting a time-series into a weighted graph (complex network), which builds on electrostatics in physics. The proposed method conceptualizes a time-series as a series of stationary, electrically charged particles, on which Coulomb-like forces can be computed. This allows generating electrostatic-like graphs associated with time-series that, additionally to the existing transformations, can be also weighted and sometimes disconnected. Within this context, this paper examines the structural similarity between five different types of time-series and their associated graphs that are generated by the proposed algorithm and the visibility graph, which is currently the most popular algorithm in the literature. The analysis compares the source (original) time-series with the node-series generated by network measures (that are arranged into the node-ordering of the source time-series), in terms of a linear trend, chaotic behaviour, stationarity, periodicity, and cyclical structure. It is shown that the proposed electrostatic graph algorithm generates graphs with node-measures that are more representative of the structure of the source time-series than the visibility graph. This makes the proposed algorithm more natural rather than algebraic, in comparison with existing physics-defined methods. The overall approach also suggests a methodological framework for evaluating the structural relevance between the source time-series and their associated graphs produced by any possible transformation.

## Introduction

The multidisciplinary nature of networks^1–3^ has introduced new directions in the time-series research that led to the emergence of the complex network analysis of time-series. This newly established research field showed a remarkable development, at a multidisciplinary level^4^, when scholars conceptualized^5–7^ that transforming a time-series into a graph can produce insights that are not visible by current time-series approaches. In general, studying the topology of a graph instead of the structure of a time-series promotes time-series analysis because it enlarges the embedding of the available information, from a first-order tensor (i.e. the time-series vector) into a second-order tensor (i.e. the graph connectivity matrix)^8^. Within this context, Zhang and Small^7^ were the first who constructed graphs from pseudo-periodic time-series, and Yang and Yang^6^ applied thresholds to the correlation matrix to convert it into a connectivity matrix. Xu et al.^9^ proposed a transformation for creating graphs from time-series based on different dynamic systems. Lacasa et al.^5^ built on the intuition of considering a time-series as a landscape and introduced a connectivity criterion based on visibility from optics physics. Gao and Zin^10^ proposed methods (i.e. flow pattern complex network, dynamic complex network, and fluid–structure complex network) to construct complex networks from experimental flow signals, and Donner et al.^11^ introduced a recurrence method converting graphs from time-series based on the phase-space of a dynamical system. Amongst the existing methods, the natural visibility graph (NVG) or, in synonym, the visibility graph algorithm (VGA) of Lacasa et al.^5^ seems to prevail in the literature either in terms of citations, or in the number of applications^12–14^, or the number of derivative methods, such as the horizontal visibility graph of Luque et al.^15^, and the visibility expansion algorithm of Tsiotas and Charakopoulos^8^,^16^. The popularity of VGA can be either due to its intuitive conceptualization from optics physics, which makes comprehension and interpretation of results easier, or to its topological consistency to convert periodic time-series to regular graphs, random time-series to random graphs, and fractal time-series to scale-free graphs. However, this method builds on a binary connectivity criterion, which leads to the development of binary connections and thus to unweighted graphs^5^. Therefore the VGA is by definition restricted in generating visibility graphs that are disassociated from the numerical scale of the source (original) time-series.

Aiming at serving the demand of promoting a weighted conceptualization in the complex network analysis of time-series, this paper introduces a method for converting a time-series into a weighted graph by using an electrostatics transformation algorithm based on Coulomb’s law. The proposed method is driven by a dual motivation: the first builds on the example of the VGA^5^, which implies that physics-defined transformations can be more intuitive and easily comprehensive than the algebraic (or computational) ones. The second one is based on the universality of Coulomb’s inverse square law^17^, which grounded the development of essential research in electromagnetism but also inspired multidisciplinary research in economics^18^, urban and spatial planning and transport engineering^19,20^, biology^21^, geophysics^22^, computational^23^ and communication sciences^24^, etc. Within this multidisciplinary context, the proposed method conceptualizes a time-series as a sequence of stationary and electrically charged particles (nodes) and generates an electrostatic graph based on pair-wise calculations of Coulomb’s law across the time-series nodes. The Coulomb-like forces are assigned as weights in the connectivity matrix of the electrostatic graph and can be seen as a measure of relevance between two nodes, in terms of the sign, scale, and spatial proximity. This approach allows quantifying the interaction between the time-series nodes and thus to conceptualize the dynamics of a time-series through the effect of electrostatic forces applied between the nodes.

The remainder of the paper is organized as follows: Sect. 2 (methods) describes the proposed ESG algorithm and its modeling context, it introduces the node-series of network measures concept in the ESG, and it briefly describes the methods used for testing the performance of the proposed algorithm. Section 3 (Results) shows the results of the multilevel analysis testing the performance of the proposed algorithm in comparison with a well-established method of converting a time-series to a graph. Finally, in Sect. 4, conclusions are given.

## Methods

### The proposed ESG algorithm

**Figure 1:**
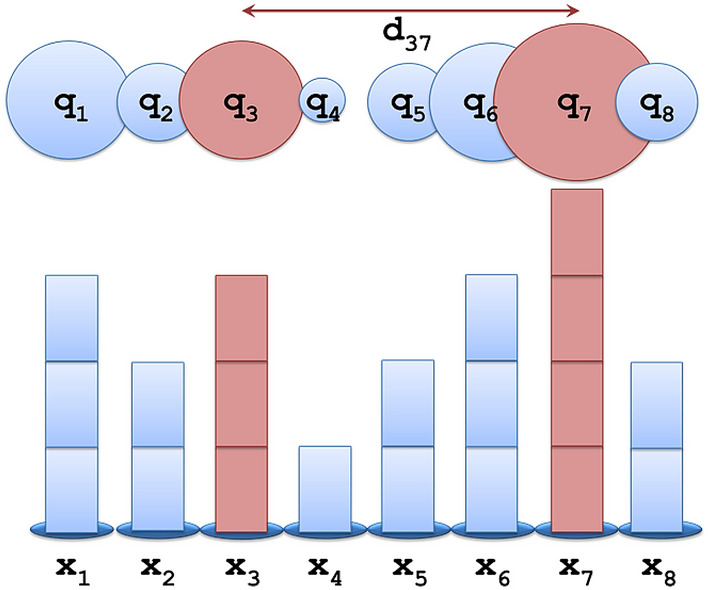
Example of the electrostatic graph (ESG) algorithm conceptualization. The volume of electrical charge (*q*_*i*_, *i* = 1,..,8) in each node is shown proportionally to the node size (*x*_*i*_, *i* = 1,..,8) of the time-series.

Let us consider a time-series *X* = {*x*_1_, *x*_2_,…, *x*_*n*_} with *n*$\in \mathbb{N}$ number of nodes *i*$\in$* X*, where each one has a real numeric value *X*(*i*) = *x*_*i*_$\in {\mathbb{R}}$. If we assume that every node *i* in the time-series can be seen as a static particle of electrical charge *q*(*i*)≡*q*_*i*_ = *x*_*i*_, we can define an (either attractive or repulsive) electrostatic force *F*_*ij*_ applied between any pair of nodes *i*,*j* (Fig. 1), according to the inverse-square Coulomb’s law expressed by the relation^17^:

1 $$F_{ij} = k_{e} \cdot \frac{{q_{i} \cdot q_{j} }}{{\left( {d_{ij} } \right)^{2} }} = k_{e} \cdot \frac{{x_{i} \cdot x_{j} }}{{\left( {d_{ij} } \right)^{2} }},$$

where *q*_*i*_ and *q*_*j*_ are the electrostatic charges of nodes *i* and *j*, *d*_*ij*_ is the intermediate discrete distance between nodes *i*,*j* that expresses steps of separation and is defined by the difference (*i*–*j*), and *k*_*e*_ is the Coulomb’s constant.

This assumption allows considering a time-series *X* as a series of stationary and electrically charged particles (i.e. time-series nodes), on which we can compute a square matrix with the Coulomb-like forces *F*(*X*) = {*F*_*ij*_ | *i*,*j* = 1, …, *n*}, according to the relation:

2 $$(1)\mathop {\mathop \Leftrightarrow \limits_{{k_{e} \equiv 1}} }\limits^{{q_{i} { = }X(i) = x_{i} }} F\left( X \right) = \{ F(X(i),X(j)) \equiv F_{{ij}} = \frac{{x_{i} \cdot x_{j} }}{{\left( {i - j} \right)^{2} }}|i,j = {\text{1}},{\text{ }} \ldots ,n\} ,$$

where *d*_*ij*_ = (*i* − *j*) and *k*_*e*_ is the Coulomb’s constant^17^, which can be considered as a scale factor and in this paper is set to *k*_*e*_ = 1.

The square structure of the *F*(*X*) matrix (with the Coulomb-like forces) can be seen as an electrostatic graph-model ESG, where each element *F*_*ij*_ $\in {\mathbb{R}}$ expresses the (attractive or repulsive) electrostatic force applied between any pair of nodes *i*,*j*. When it is important to note that the ESG is associated with the time-series *X*, we can symbolize the electrostatic graph as ESG(*X*). In terms of graph theory^25^, *F*(*X*) is the weighted connectivity matrix of an undirected graph *G*_*ESG*_(*V*,*E*), where *V* is the node-set and *E* is the edge-set. The weights (*w*_*ij*_) in the ESG’s weighted connectivity matrix are equal to the Coulomb-like forces (*w*_*ij*_ = *F*_*ij*_) and can be seen as a measure of similarity between two nodes, in terms of the sign, scale, and spatial proximity. In particular, positive weights (*w*_*ij*_ > 0) indicate that nodes *i*,*j* have homogeneous arithmetic signs, where negative cases (*w*_*ij*_ < 0) imply that they have heterogeneous signs. Also, high *w*_*ij*_ scores may imply either that nodes *i*,*j* are close in the time-series line, in terms of spatial proximity, or that they have relatively high arithmetic values or both. Within the context of the electrostatic conceptualization, the attraction expressed by a negative Coulomb-like force (*w*_*ij*_ = –*F*_*ij*_) can be seen as a tendency of the nodes to balance their heterogeneity and converge toward the horizontal axis, whereas the repulsion expressed by a positive force (*w*_*ij*_ =  + *F*_*ij*_) can be seen as a tendency of the nodes to escape from their homonymous electrostatic balance and thus to evolve (either increasingly or decreasingly) through time.

By definition, Coulomb’s law determines a field of infinite distance, where the electrostatic forces are noticed at infinity, although they are negligible. This property makes the ESG by default a fully connected (complete) graph *K*_*n*_, namely a graph where all nodes are linked to any other. Provided that a complete graph *K*_*n*_ has a trivial topology, in terms of complexity (since the average degree is always $\left\langle k \right\rangle$ = *n*–1 and most of other metrics, such as average path length, network diameter, graph density, and clustering coefficient are equal to one), we filter the set *E* of the ESG connections, aiming to generate more complex topologies of electrostatic graphs. In particular, we consider a threshold *F*_*c*_, defined within the interval $F_{c} \in \left( {\min \left\{ {F_{ij} } \right\},\max \left\{ {F_{ij} } \right\}} \right)$, so that the weighted connectivity matrix *W*_*ESG*_ include those values that are equal or above *F*_*c*_, as it is expressed by the relation:

3 $$W_{ESG} = \{ F_{ij} \ne 0 \in F(X):F_{ij} \ge F_{c} \} \subseteq F(X),$$

where *F*(*X*) is the Coulomb-like matrix defined in relation (1). This filtering allows considering numerous electrostatic graphs ESG(*X*), which are expressed as a function *W*_*ESG*_ = *f*(*F*_c_) of the threshold-variable *F*_*c*_. To introduce a reference value to the threshold-variable *F*_*c*_, we define a typical value *f*_*z*_ by the relation:

4 $$F_{c} (f_{z} ) = f_{z} = \frac{1}{{n - {1}}} \cdot \sum\limits_{n} {x_{n} } = \frac{n}{n - 1} \cdot \left\langle x \right\rangle = n \cdot {\text{sgn}} \left( {\left\langle x \right\rangle } \right) \cdot \frac{{\sqrt {\left| {\left\langle x \right\rangle } \right|} \cdot \sqrt {\left| {\left\langle x \right\rangle } \right|} }}{{\left( {\sqrt {n - {1}} } \right)^{2} }},$$

where *n* is the number of time-series nodes, $\left\langle \cdot \right\rangle$ is the average operator, and sgn(·) is the sign (or signum) function^26^. In numeric terms, the *f*_*z*_ filtering describes that non-zero elements of the weighted ESG’s connectivity matrix are those with values higher than the adjusted mean-value $\frac{n}{n - 1} \cdot \left\langle x \right\rangle$ of the time-series *X*. In physical (electrostatic) terms, *f*_*z*_ describes an electrostatic force that is *n*-times greater than this applied to a pair of particles with electrical charges *q*_*i*_, *q*_*j*_ = $\sqrt {\left| {\left\langle x \right\rangle } \right|}$(i.e. equal to the square-root of the absolute mean-value of the time-series *X*), which are *d*_*ij*_ = $\sqrt {n - {1}}$ (i.e. equal to the square-root of the time-series length) steps of separation distant.

**Figure 2:**
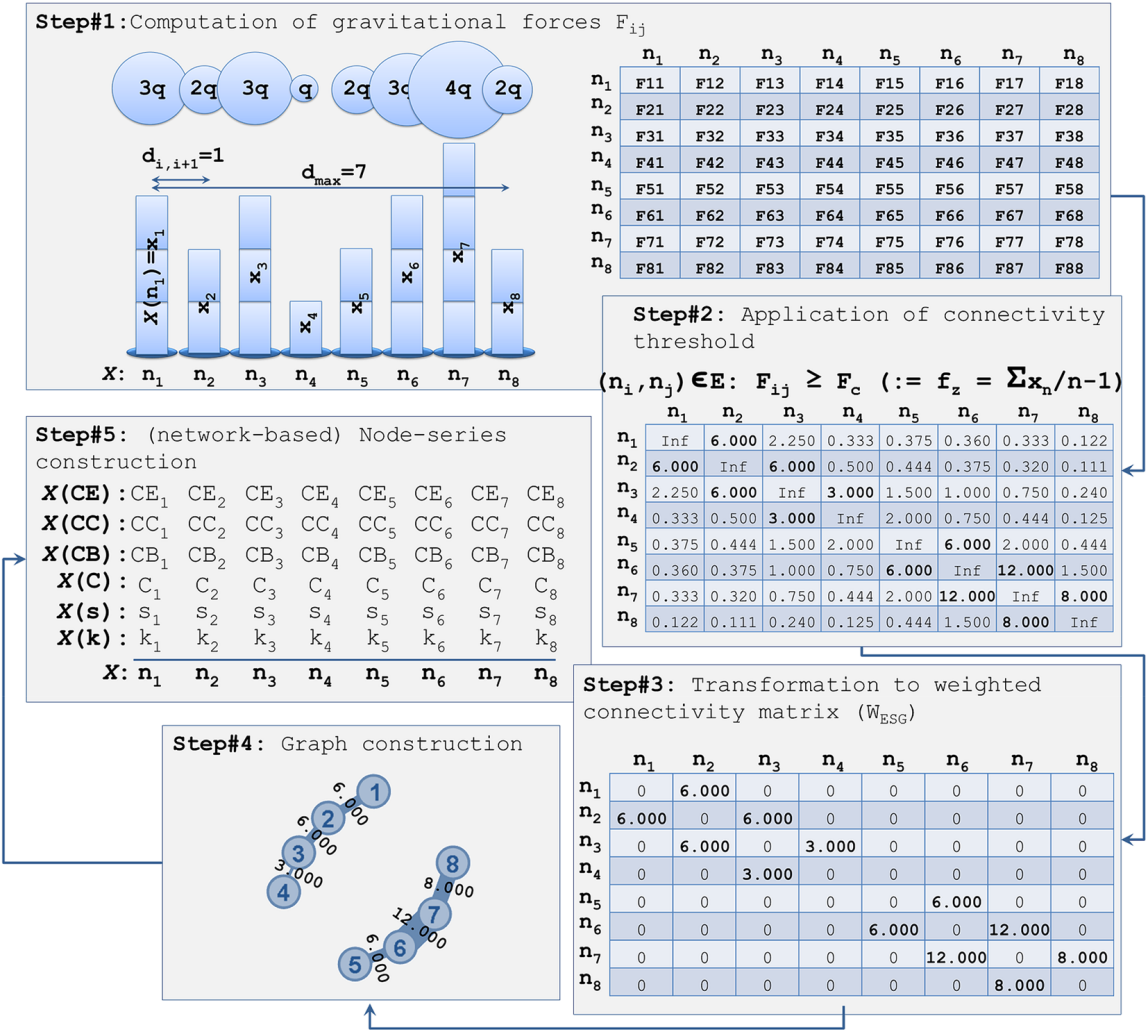
The methodological framework of the study. Steps #1–#4 describe the ESG algorithm generating an electrostatic graph from a time-series *X* = {*x*_*i*_ | *i* = 1, …, 8}. Step#5 describes the process of generating secondary time-series from the network measures of ESG(*X*).

Within this context, the proposed ESG algorithm is implemented in four steps, as it is shown in Fig. 2. First, we compute the matrix *F*(*X*) of Coulomb-like forces, according to the relations (1) and (2). Secondly, we apply to *F*(*X*) the connectivity filter and compute the weighted connectivity matrix *W*_*ESG*_, according to the relations (3) and (4). Thirdly, we manage the disconnected data (i.e. mainly the diagonal element yielding infinite computations due to zero distances included in the denominator) of *F*(*X*), by substituting “inf” (infinite) values by zeros. Fourthly, we create the graph-layout of the ESG(*X*) based on the weighted connectivity matrix *W*_*ESG*_.

According to the first four steps of the algorithm, we can generate the electrostatic graph ESG(*X*), which is associated with a time-series *X* and is an undirected and weighted graph with a non-trivial topology. In this graph model, we can further compute several network measures and metrics and thus reveal the topological properties of the ESG. Therefore, at the fifth and final step of the algorithm, we compute node-series of network measures of the ESG(*X*), and afterward, we compare their structural relevance with this of the source time-series *X*. The procedure is described in more detail in the following paragraphs.

### Node-series of network measures

The electrostatic graph ESG(*X*) is a graph-model *G*_*ESG*_(*V*,*E*) where each network node *v*_*i*_ $\in$ *V* is the same with a time-series node *i* $\in$ *X*, namely *v*_*i*_≡*i* $\in$ *V*,*X*. Therefore, for every node-measure *Y* (e.g. node degree, local clustering coefficient, closeness, betweenness, and eigenvector centrality, etc.) of the ESG, we can arrange the scores *Y*(*v*_*i*_) = *y*_*i*_ into the time-series *X* = {*x*_1_, *x*_2_,…, *x*_*n*_} ordering, and thus to configure node-series *X*(*Y*) = {*y*_1_, *y*_2_,…, *y*_*n*_} of the ESG network measures that are associated with the source time-series. This allows comparing the source time-series *X* with these of the ESG node-series *X*(*Ys*) and detecting possible structural similarities that can be seen as a measure of relevance between the time-series and the ESG. The available network (node) measures that participate in the construction of the node-series are shown in Table 1.

**Table 1:** The node measures that are considered in the analysis ^1,27-29^

Measure	Description	Mathematical Expression
*Node Degree* (*k*)	The number of edges being adjacent to a node *i*	$$\begin{gathered} k_{i} = k(i) = \sum\limits_{j \in V} {\delta_{ij} } ,{\text{ where}} \hfill \\ \, \delta_{ij} = \left\{ {\begin{array}{*{20}c} {1,{\text{ if }}e_{ij} \in E} \\ {0,{\text{ otherwise}}} \\ \end{array} } \right. \hfill \\ \end{gathered}$$
*Node strength (s)*	The sum of edge weights being adjacent to a given node *i*	$$\begin{gathered} s_{i} = s(i) = \sum\limits_{j \in V(G)} {\delta_{ij} \cdot w_{ij} } , \hfill \\ {\text{where }}w_{ij} = w(e_{ij} ) \hfill \\ \end{gathered}$$
*Local Clustering Coefficient* (*C*)	The number of a node’s connected neighbors *E*(*i*), divided by the number of the total triplets *k*_*i*_(*k*_*i*_–1) shaped by the node *i*	$C(i) = \frac{E(i)}{{k_{i} \cdot \left( {k_{i} - 1} \right)}}$
*Closeness Centrality* (*CC*)	Total binary distance *d*(*i*,*j*) computed on the shortest paths originating from a given node *i* and having destination all the other nodes *j* in the network. This measure expresses the node’s reachability in terms of steps of separation	$CC(i) = \frac{1}{n - 1} \cdot \sum\limits_{j = 1,i \ne j}^{n} {d_{ij} } = \overline{d}_{i}$
*Betweenness Centrality* (*CB*)	Fraction of all shortest paths *σ*(*i*) including a given node *i*, to the number *σ* of all the shortest paths in the network	$CB(i) = {{\sigma (i)} \mathord{\left/ {\vphantom {{\sigma (i)} \sigma }} \right. \kern-\nulldelimiterspace} \sigma }$
*Eigenvector Centrality* (*CB*)	Spectral measure expressing the influence of node *i* in the network. In the formula *N*(*i*) expresses the neighborhood of node *i*, *a*_*ij*_ an element of the adjacency, *x*_*j*_ the j-th component of the adjacency’s eigenvector with eigenvalue equal to *λ*	$CE(i) = \frac{1}{\lambda } \cdot \sum\limits_{j \in N(i)} {a_{ij} \cdot x_{j} }$

In terms of notation, for a (source) time-series *X* = {*x*_1_, *x*_2_,…, *x*_*n*_}, where *n*$\in {\mathbb{N}}$ and *x*_*i*_$\in {\mathbb{R}}$, we can write its associated node-series for the network measure *Y* as *X*(*Y*) = {*Y*(*x*_1_), *Y*(*x*_2_),…, *Y*(*x*_*n*_)} = {*y*_1_, *y*_2_,…, *y*_*n*_}. We can read that *X*(*Y*) is “the node-series of the network measure *Y*, which is computed for the ESG that is associated with the time-series *X*” or, in brief, that *X*(*Y*) is “the node-series of (the measure) *Y* for the ESG”. Within this context, we can compute the node-series for the measures of degree *X*(*Y* = *k*) = {*k*_1_, *k*_2_,…, *k*_*n*_}, strength *X*(*s*) = {*s*_1_, *s*_2_,…, *s*_*n*_}, clustering coefficient *X*(*C*) = {*C*_1_, *C*_2_,…, *C*_*n*_}, betweenness centrality *X*(*CB*) = {*CB*_1_, *CB*_2_,…, *CB*_*n*_}, closeness centrality *X*(*CC*) = {*CC*_1_, *CC*_2_,…, *CC*_*n*_}, and eigenvector centrality *X*(*CE*) = {*CE*_1_, *CE*_2_,…, *CE*_*n*_}, according to the mathematical formulas shown in Table 1. Provided that we can generate a node-series for any graph *G*(*X*) that is associated with a time-series *X*, we can include a subscript index in the notation *X*_*G*_(*Y*) when necessary (e.g. *X*_*ESG*_(*k*)) to denote the type of graph that the time-series is associated with.

### The effect of the connectivity threshold on the ESG topology

**Figure 3:**
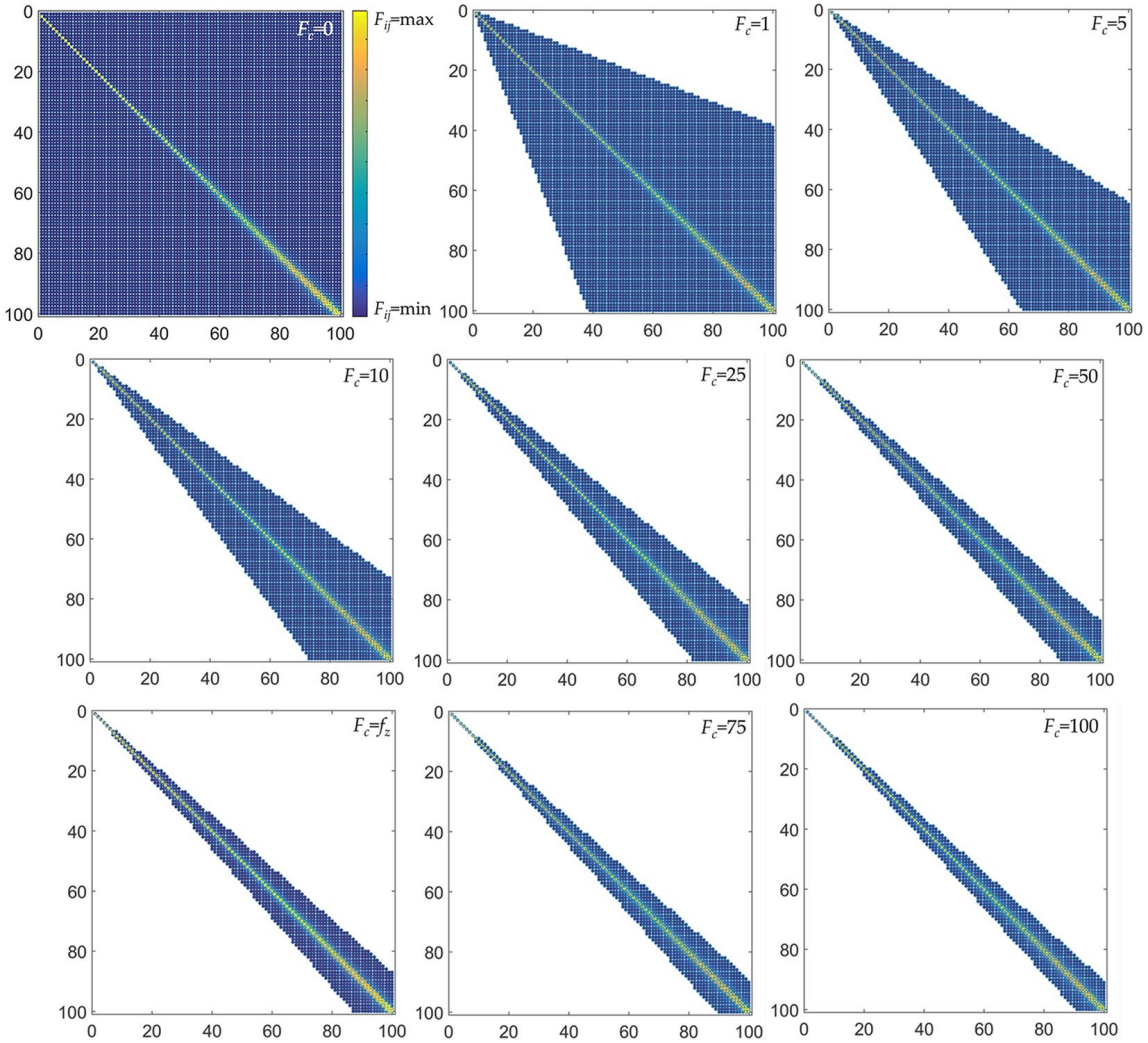
Sparsity (spy) plots of the ESGs that are associated with the series *X*_1:100_ = {1, 2,…, 100} and are computed for the connectivity thresholds *F*_*c*_ = 0, *F*_*c*_ = 1, *F*_*c*_ = 5, *F*_*c*_ = 10, *F*_*c*_ = 25, *F*_*c*_ = 50, *F*_*c*_ = *f*_*z*_, *F*_*c*_ = 75, and *F*_*c*_ = 100.

The connectivity threshold *F*_*c*_ that is applied to the Coulomb-like matrix, according to relation (3), is determinative for the configuration of the ESG topology. To illustrate this, let us consider the series *X*_1:100_ = {1, 2,…, 100} of the first hundred natural numbers. By applying to this series sequentially the connectivity thresholds *F*_*c*_ = 0, *F*_*c*_ = 1, *F*_*c*_ = 5, *F*_*c*_ = 10, *F*_*c*_ = 25, *F*_*c*_ = 50, *F*_*c*_ = *f*_*z*_, *F*_*c*_ = 75, and *F*_*c*_ = 100, we get various ESGs, as it is shown in Fig. 3.

**Figure 4:**
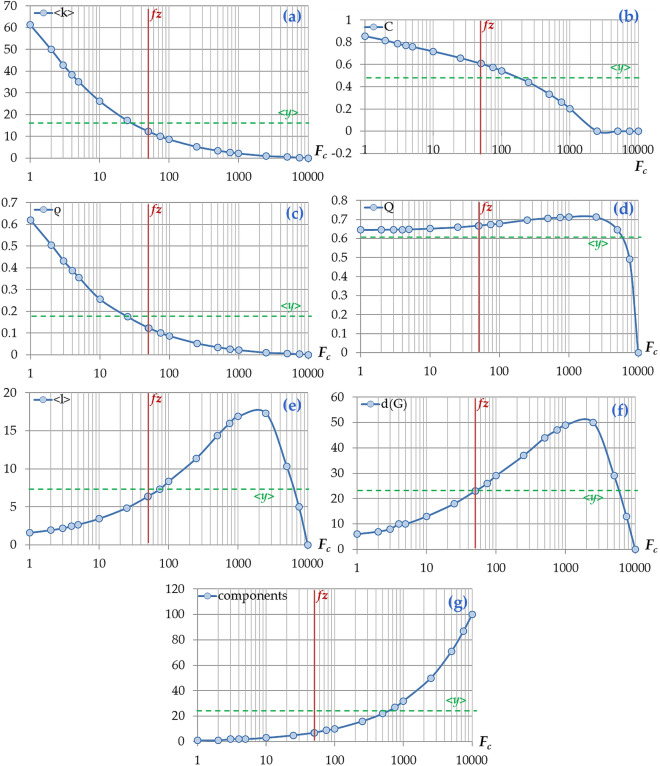
**.** Line diagrams showing how the network measures of (**a**) average node degree $\left\langle k \right\rangle$, (**b**) clustering coefficient *C*, (**c**) graph density *ρ*, (**d**) modularity *Q*, (**e**) average path length $\left\langle l \right\rangle$, (**f**) network diameter d(*G*), and (g) number of components change as a function of the connectivity threshold *F*_*c*_, for a series *X*_1:100_ = {1, 2,…, 100}, where *F*_*c*_ranges within the interval *F*_*c*_ $\in$[0, max{ *X*_1:100_}^2^]. The bold vertical line represents the typical value *f*_*z*_ defined at relation (4), whereas the dashed horizontal line a point estimate of the average *y* values.

As it can be observed, the ESGs shown in Fig. 3 appear quite different in terms of graph density and node arrangement in the adjacency matrix. In particular, as the *F*_*c*_ becomes greater, the connectivity strip toward the main diagonal in the adjacency matrix becomes narrower, expressing each time a separate connectivity pattern. To examine whether and how the network topology is affected by changes in *F*_*c*_, we compute a set of network measures and metrics (average degree $\left\langle k \right\rangle$, clustering coefficient *C*, graph density *ρ*, modularity *Q*, average path length $\left\langle l \right\rangle$, network diameter d(*G*), and the number of components) for a series of ESGs that are generated by applying connectivity thresholds ranging within the interval *F*_*c*_$\in$[0, *n*^2^ = max{ *X*_1:100_}^2^ = 10^4^]. This approach assumes that the network topology is collectively approximated by the set of available network measures, where each measure represents a certain topological aspect. The results of the analysis are shown in Fig. 4, where each network measure is expressed as a function of the connectivity threshold *F*_*c*_.

Also, is evident that all network measures considerably fluctuate as the connectivity threshold *F*_*c*_ changes. The cases of average degree $\left\langle k \right\rangle$(Fig. 4a), clustering coefficient *C* (Fig. 4b), and graph density *ρ* (Fig. 4c) follow a declining pattern to the changes of *F*_*c*_, the cases of average path length $\left\langle l \right\rangle$(Fig. 4e) and graph density d(*G*) (Fig. 4f) follow a bell-shaped pattern of negative skew (asymmetry), whereas the number of components (Fig. 4g) follows an increasing pattern. For the case of modularity *Q* (Fig. 4d), the performance of this measure appears considerably invariant along the biggest part of the *F*_*c*_’s interval. As far as the typical value *f*_*z*_ is concerned, we can observe that this value cannot be related to border (i.e. min or max) distribution values, but it can be quite indicative of the average performance of the topological aspects of the ESGs. This indication can support the goodness of the choice of defining the typical *f*_*z*_ value within a physical (Coulomb-like) context, as it is shown in relation (4).

Overall, this analysis shows that the choice of the connectivity threshold *F*_*c*_ can be determinative to the topological features and generally the topology of the resulting ESG. This observation is evident even by the examination of a simple linear series of natural ascending numbers, which can only be considered as an indicative approach for the ESG construction. However, even this simple consideration sufficed to highlight the dependence between the connectivity threshold and the ESG’s network topology and thus to introduce a methodological path for optimally defining the *F*_*c*_ value. The examination of the optimum or most representative threshold is a matter of specialized optimization analysis that introduces avenues of further research and falls outside the scope of this paper. However, the physically defined approaches, as this of the Coulomb-like definition of the *F*_*c*_ shown in relation (4), or others utilizing methods from other disciplines can become insightful toward this optimization direction and are suggested for further research promoting multidisciplinary conceptualization. For instance, further research on this topic can apply to different types of time-series and more thorough optimization analysis in the choice of *F*_*c*_. For the scope of this paper, the choice of the typical value *f*_*z*_ for the connectivity threshold is considered satisfactory to provide a reference value that is representative of the average topological features of the ESGs.

### Testing the performance of the ESG algorithm

**Figure 5:**
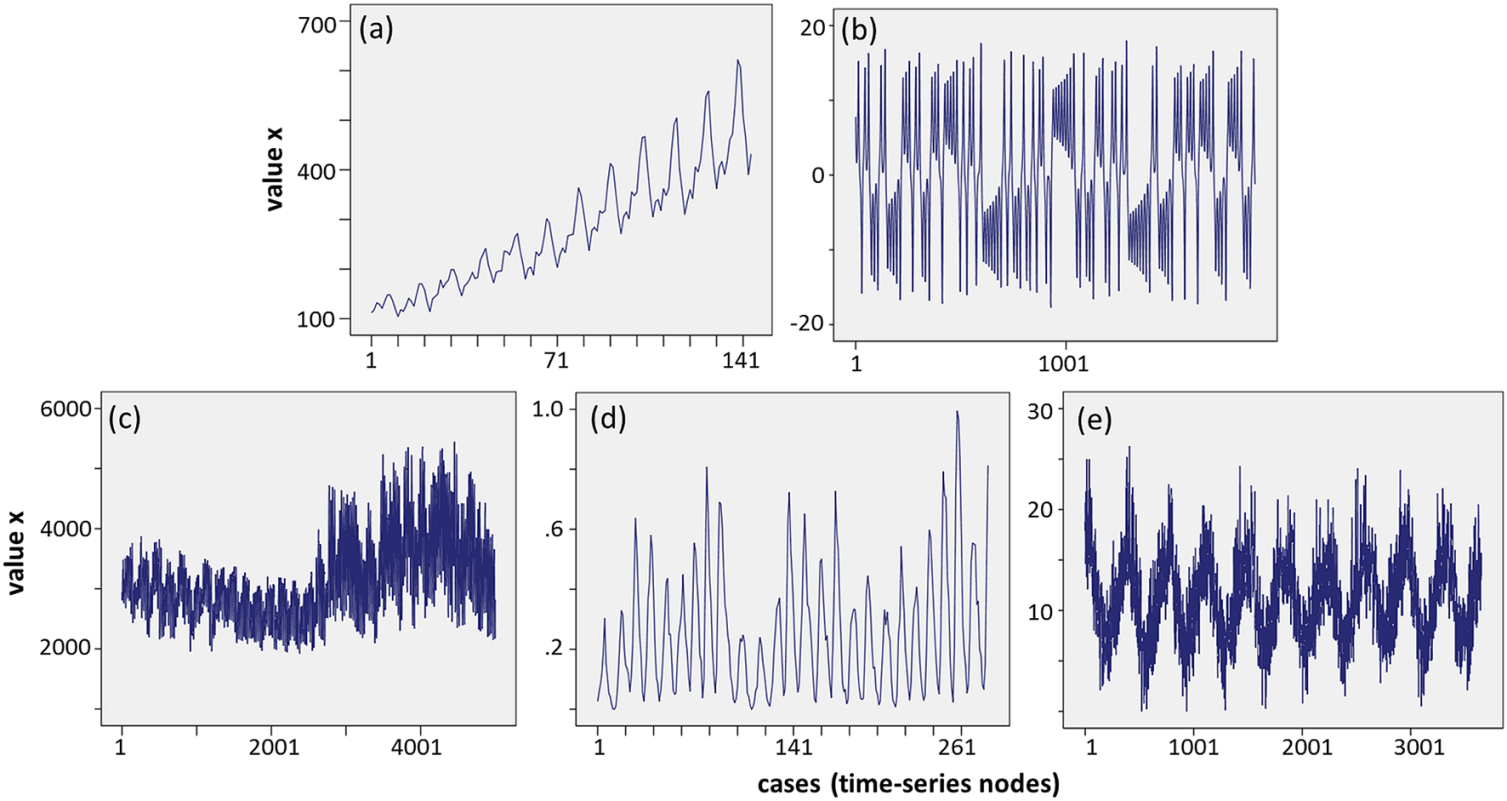
The source (reference) time-series considered in the analysis represent distinctive different patterns, where (**a**) is an air-passengers time-series with linear trend (*X*_*a*_: 144 cases, including the monthly totals of a US airline passengers for the period 1949 to 1960), (**b**) is the typical Lorentz chaotic time-series (*X*_*b*_: 1900 cases, created from the Lorenz equations, on standard values sigma = 10.0, r = 28.0, and b = 8/3), (**c**) is a part (*X*_*c*_: 5000 cases) of a broader stationary time-series including estimated energy consumption, in Megawatts (MW), for the Duke Energy Ohio/Kentucky, (**d**) is a periodical time-series (*X*_*d*_: 280 cases, including wolfer sunspot numbers for the period 1770 to 1771), and (**e**) is a cyclic time-series (*X*_*e*_: 3650 cases, including daily minimum temperatures in Melbourne, Australia, for the period 1981–1990).

The analysis examines five different types of time-series, as it is shown in Fig. 5. The first one (Fig. 5a) was extracted from AirPassengers^30^ and is a time-series with a linear trend (abbreviated: *X*_*a*_≡AIR), including the monthly totals of US airline passengers for the period 1949 to 1960 (144 cases). The second one (Fig. 5b) was extracted from LorentzTS^31^ and is a typical Lorentz chaotic time-series (*X*_*b*_≡CHAOS) generated from the Lorenz differential equations, on standard values sigma = 10.0, r = 28.0, and b = 8/3. This time-series has a length of 1900 cases. The third one (Fig. 5c) was extracted from DEOK.hourly^32^ and is a part (the first 5000 cases) of a broader stationary time-series (of 57,739 cases) including estimated energy consumption, in Megawatts (MW), for the Duke Energy Ohio/Kentucky (*X*_*c*_≡DEOK). Next, the fourth one (Fig. 5d) was extracted from Wolfer-sunspot-numbers^33^ and is a periodic time-series including Wolfer sunspot numbers (*X*_*d*_≡SUNSPOTS), for the period 1770 to 1771 (280 cases). The fifth one (Fig. 5e) was extracted from Daily-minimum-temperatures-in-me^34^ and is a cyclical time-series including daily minimum temperatures in Melbourne, Australia (*X*_*e*_≡TEMP), for the period 1981–1990 (3650 cases). Links to the time-series databases are available in the reference list.

To examine the effectiveness of the proposed algorithm, we firstly compare the structure of the source time-series *X* with its node-series *X*_*ESG*_(*Ys*) of the ESG node measures (*Ys*). Such comparisons are driven by the rationale that the ESG is a transformation (conversion) of a time-series to a complex network and therefore possible similarities that can be detected in the structural properties (e.g. data variability, linear trend, chaotic, stationary, periodic, and cyclical structure) between the original time-series and its associated ESG node-series can be seen as aspects of homeomorphism describing this transformation. In general, this approach is expected to illustrate the level at which the topology of the associated electrostatic graph ESG(*X*) sufficiently incorporates structural information of the source time-series *X*. Secondly, we compare the structure of the *X*_*ESG*_(*Ys*) node-series with this of their concordant node-series *X*_*VGA*_(*Ys*) of the node measures (*Ys*) computed in the visibility graphs defined by Lacasa et al.^5^. The comparisons between the source time-series and its associated node-series (either of ESG or VGA conversion) build on a multilevel analysis consisting of five tests; the first one detects similarities in data-variability (i.e. whether the original time-series and the node-series have the same fluctuation patterns) based on the Pearson’s bivariate coefficient of correlation^35,36^, the second one in linear-trend by using the Linear Regression (LSLR) fitting^36^, the third one in chaotic-structure based on the correlation dimension and embedding dimension diagram^37^, the fourth one in stationary-structure based on the augmented Dickey-Fuller test (ADF) for a unit root ^38^, and the fifth one in periodic-structure based on autocorrelation function^38^. Each test is briefly described in the following paragraphs.

### The visibility graph algorithm

**Figure 6:**
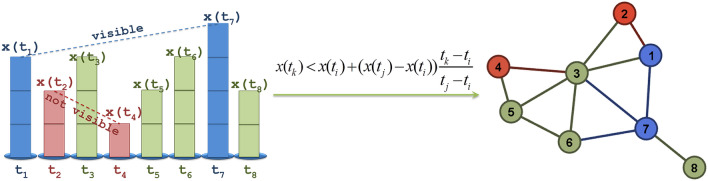
(left) Example a pair of visible (shown in blue colour) and another of not visible (shown in red colour) time-series nodes (generally shown in green colour) defined according to the natural visibility algorithm (NVG), (right) the visibility graph generated from the time-series shown at the left side.

The natural visibility algorithm (NVG) was proposed by Lacasa et al.^5^ and builds on the intuition of considering a time-series as a path of successive mountains of different height, where each represents the value of the time-series at a certain time. In this time-series landscape, an “observer” standing on the top of a mountain can see (either forward or backward) as far as possible, provided that no other top obstructs its visibility field (Fig. 6).

In mathematical terms, each time-series node (*t*_*i*_, *x*(*t*_*i*_)) corresponds to a graph node *i≡*(*t*_*i*_, *x*(*t*_*i*_))$\in$ *V*, and thus two nodes *i*,*j* $\in$ *V* are connected (*i*,*j*)$\in$ *E* in the visibility graph when the following inequality (NVG connectivity criterion) is satisfied:

6 $$X(t_{k} ) < X(t_{i} ) + (X(t_{j} ) - X(t_{i} ))\frac{{t_{k} - t_{i} }}{{t_{j} - t_{i} }}, \, \forall k \in (i,j){ ,}$$

where *X*(*t*_*i*_) and *X*(*t*_*j*_) are the numerical values of the time-series nodes (*t*_*i*_, *x*(*t*_*i*_))*≡i* and (*t*_*j*_, *x*(*t*_*j*_))*≡j* and *t*_*i*_, *t*_*j*_ express their time points. In geometric terms, a visibility line can be drawn between two time-series nodes *i*,*j* $\in$ *V*, if no other intermediating node (*t*_*k*_, *x*(*t*_*k*_))*≡k* obstructs their visibility. That is, two time-series nodes are connected in the visibility graph whether no other intermediary node is higher so that to intersect the visibility line defined by this pair of nodes (Fig. 6). Therefore, two time-series nodes can enjoy a connection in the associated visibility graph if they are visible through a visibility line. The visibility algorithm conceptualizes the time-series as a landscape and generates a visibility graph associated with this landscape. The associated (to the time-series) visibility graph is a complex network where complex network analysis can be further applied^8,16^.

### Correlation analysis

At the first step of the analysis, we detect linear correlations between the source time-series *X* and the available (ESG and VGA) node-series. This approach examines whether the original time-series *X* and the node-series {*X*_*i*_(*k*), *X*_*i*_(*s*), *X*_*i*_(*C*), *X*_*i*_(*CB*), *X*_*i*_(*CC*), and *X*_*i*_(*CE*) | *i* = ESG,VGA} have the same fluctuation patterns and thus they can be considered as relevant in terms of data variability. In this analysis, the Pearson’s bivariate coefficient of correlation^35,36^ is used, which ranges at the interval *r*_*X*,*Y*_ $\in$[–1,1] and detects linear (either positive or negative) correlations when $\left| {r_{XY} } \right| \to 1$.

### Test of the linear trend

To detect a linear trend, we apply linear fittings to the source time-series *X* and to its associated node-series { *X*_*i*_(*k*), *X*_*i*_(*s*), *X*_*i*_(*C*), *X*_*i*_(*CB*), *X*_*i*_(*CC*), and *X*_*i*_(*CE*) | *i* = ESG,VGA}. According to this approach^36^, a linear curve $\hat{y} = b \cdot f(x) + c$ is fitted to the available data that bests describes their variability. The curve fitting algorithm estimates the parameters *b, c* minimizing the square differences $y_{i} - \hat{y}_{i}$^36^, according to the relation:

7 $$\min \left\{ {e = \mathop \sum \limits_{i = 1}^{n} \left[ {y_{i} - \hat{y}_{i} } \right]^{2} = \mathop \sum \limits_{i = 1}^{n} \left[ {y_{i} - \left( {\mathop \sum \nolimits b_{i} f_{i} (x) + c} \right)} \right]^{2} } \right\},$$

where *y*_*i*_ express the observed and $\hat{y}_{i}$ the estimated values. The optimization method that is used is the Least-Squares Linear Regression (LSLR) method^36^, which assumes that the differences $e = \mathop \sum \limits_{i = 1}^{n} \left[ {y_{i} - \hat{y}_{i} } \right]^{2}$ follow the normal distribution *e* ~ *N*(0,$\sigma_{e}^{2}$). The goodness of the model fit is measured by the coefficient of determination *R*^2^, which is defined by the expression^35,36^:

8 $$R^{2} = {{\left( {\mathop \sum \nolimits_{i = 1}^{n} \left( {\hat{y}_{i} - \overline{y}} \right)^{2} } \right)} \mathord{\left/ {\vphantom {{\left( {\mathop \sum \nolimits_{i = 1}^{n} \left( {\hat{y}_{i} - \overline{y}} \right)^{2} } \right)} {\left( {\mathop \sum \nolimits_{i = 1}^{n} \left( {y_{i} - \overline{y}} \right)^{2} } \right)}}} \right. \kern-\nulldelimiterspace} {\left( {\mathop \sum \nolimits_{i = 1}^{n} \left( {y_{i} - \overline{y}} \right)^{2} } \right)}},$$

where $\overline{y}$ is the average of the observations and *n* is the number of cases (i.e. the series length). The coefficient of determination expresses the amount of variability of the response variable that is expressed by the linear model and ranges within the interval [0,1], indicating perfect linear determination when *R*^2^ = 1^35,36^. Within this context, amongst the ESG and VGA node-series, those being closer to the source time-series *X* in determination and model configuration (i.e. values in *b* and *c* estimators) are considered as more relevant to *X* in terms of linear trend.

### Detection of chaotic structure

To detect chaotic structure in a time-series, we examine the patterns of the correlation (*v*) versus the embedding dimension (*m*) scatter plots (*v*,*m*). According to the Chaos theory^37^, the correlation dimension (*v*) is a measure of the dimensionality of the space occupied by a set of random points and thus is used to determine the dimension of the fractal objects, which is often called fractal dimension. For a time-series *X* = {*x*_*i*_ | *i* = 1, …, *n*}, the correlation integral *C*(*ε*) is calculated by the expression^39,40^:

9 $$C\left( \varepsilon \right) = \mathop {\lim }\limits_{n \to \infty } \frac{N(\varepsilon )}{{n^{2} }}\sim \varepsilon^{v} ,$$

where *N*(*ε*) is the total number of pairs of time-series points (*x*_*i*_, *x*_*j*_) with a distance smaller than ε, namely *d*(*x*_*i*_,*x*_*j*_) = *d*_*ij*_ < *ε*. As the number of points tends to infinity (*n* → ∞), and therefore as their corresponding distances tend to zero (*d*_*ij*_ → 0), the correlation integral tends to the quantity *C*(*ε*) ~ *ε*^*v*^, where *v* is the so-called correlation dimension. Intuitively, the correlation dimension expresses the ways to which the points can be close to each other along different dimensions and is expected to rise faster when the space of embedding is of a higher dimension. Therefore, the correlation (*v*) versus the embedding dimension (*m*) diagram (*v*,*m*) can provide insights into how the time-series points are close to each other, as the dimensionality of the space of embedding increases^39,40^. Within this context, amongst the ESG and VGA node-series, those with the (*v*,*m*) diagram being closer to the source time-series *X* are considered as more relevant to the original time-series, in terms of chaotic structure.

### Detection of stationarity

To detect stationarity in the available series we apply the augmented Dickey-Fuller test (ADF) for a unit root ^38^. The ADF algorithm examines the null hypothesis (*H*_o_) that a unit-root is present in the model’s time-series data, which is expressed by the relation:

10 $$y_{t} = c + \delta {\text{t + }}\phi \cdot y_{t - 1} + \beta_{1} \cdot \Delta y_{t - 1} + \cdots + \beta_{p} \cdot \Delta y_{t - p} + \varepsilon_{t} ,$$

where Δ is the differencing operator (Δ*y*_*t*_ = *y*_*t*_ − *y*_*t*−1_), *p* is the number of lagged difference terms (specified by the user), *c* is a drift term, ***δ*** is a deterministic trend coefficient, $\phi$ is an autoregressive coefficient, *β*_*i*_ are the regression coefficients of the lag differences, and *ε*_*t*_ is a mean zero innovation process. According to Eq. (10), the unit-root hypothesis testing is expressed as follows^38^:

11 $$H_{o} :\phi = {\text{1 vs}}{. }H_{1} : \phi < {1,}$$

and the (lag adjusted) test statistic *DF* is defined by the expression^38^:

12 $$DF = \frac{{N(\overset{\lower0.5em\hbox{$\smash{\scriptscriptstyle\frown}$}}{\phi } - 1)}}{{(1 - \overset{\lower0.5em\hbox{$\smash{\scriptscriptstyle\frown}$}}{\beta }_{1} - ... - \overset{\lower0.5em\hbox{$\smash{\scriptscriptstyle\frown}$}}{\beta }_{p} )}},$$

where the uppercase symbol ‘^’ expresses an estimator. Within this context, amongst the node-series of ESG and VGA, first those satisfying the null hypothesis and then those that have more similar *DF* statistics with the source time-series *X* are considered as more relevant to the original time-series, in terms of stationarity.

### Detection of periodicity and cyclical structure

To detect periodicity in the available time-series we use the autocorrelation function (ACF), which is defined as:

13 $$\rho (s,t) = \frac{{\gamma_{x} (s,t)}}{{\sqrt {\gamma_{x} (s,s)\gamma_{x} (t,t)} }},$$

where (*s*,*t*) are time points and *γ*_*x*_(*s*,*t*) is the autocovariance function of the variable *x*^38^. In general, the ACF measures the linear predictability of the series at time *t* by using only the value *x*_*s*_ (at time *s*) with a time-lag d*t* = *t* − *s*. The ACF lies within the interval − 1 ≤ *ρ*(*s*,*t*) ≤ 1, where positive coefficient values imply a positive linear trend and negative values a negative one. Based on the ACF, we construct a set of ACF-variables, where the first refers to the source time-series *X* and the others to the node-series *X*_*i*_(*k*), *X*_*i*_(*s*), *X*_*i*_(*C*), *X*_*i*_(*CB*), *X*_*i*_(*CC*), and *X*_*i*_(*CE*), where index *i* can be either *i* = ESG or VGA. Each variable includes 30 elements corresponding to ACFs of lag d*t* = 1,2,…,30, respectively, namely:

14 $$ACF(X) = \left\{ {\rho (t,t + 1),\rho (t,t + 2),...,\rho (t,t + 30)} \right\}.$$

By constructing these *ACF*-variables, we compute the Pearson’s bivariate coefficient of correlation^35,36^ to detect linear correlations between the *ACF*(*X*) variable of the source time-series *X* and the other node-series variables. Within this context, amongst the available ESG and VGA node-series variables, those being higher correlated with the source time-series *X* are considered as more relevant to the original time-series in terms of periodicity and cyclical (i.e. periodic with a constant oscillation height) structure.

## Results

### Spy plots and graph layouts

The spy plots and graph layouts of the ESG(*X*) and VGA(*X*) graphs associated with the time-series *X* are shown in Fig.A1-A5 (in the Appendix). The spy plots are matrix-plots displaying with dots the non-zero elements of the adjacency matrix and they can thus represent the graph topology within the matrix-space^3,41^. On the other hand, network visualization is implemented by using the “Force-Atlas” layout, which is available in the open-source software of Bastian et al.^42^. This layout is generated by a force-directed algorithm, which applies repulsion strengths between network hubs while it arranges hubs’ connections into surrounding clusters. Graph models that are represented in this layout have therefore their hubs centered and mutually distant (i.e. intermediate distance between hubs is the highest possible), whereas lower-degree nodes are placed as closely as possible to their hubs^3^.

As it can be observed in Fig.A1 (Appendix), the ESG(*X*_*a*_) spy plot has a connectivity pattern configuring a tie (along the main diagonal) of increasing width (Fig.A1.a,c, Appendix), which appears indicative of the increasing trend of the source time-series (*X*_*a*_ = AIR). An aspect of such trend is also evident in the chain-like ESG(*X*_*a*_) graph layout (Fig.A1.e, Appendix), where a cluster of hubs appears on the right side that resembles the tie configuration shaped in the spy plot. Also, the saw-like pattern of the source time-series appears smoother in the pattern of the 2d ESG(*X*_*a*_) spy plot (Fig.A1.a, Appendix), whereas is more evident in the diagonal arrangement of the 3d ESG(*X*_*a*_) spy plot (Fig.A1.c, Appendix). On the other hand, the VGA(*X*_*a*_) spy plot configures a periodic pattern (Fig.A1.b,d, Appendix), where no linear trends are visible. This can be also observed in the VGA(*X*_*a*_) graph layout (Fig.A1.f, Appendix), which shapes an almost symmetric hub-and-spoke pattern.

In Fig.A2, the ESG(*X*_*b*_) spy plot configures a fractal-like tiling (Fig.A2.a, Appendix) illustrating a chaotic structure. Although such structure in the ESG(*X*_*b*_) graph layout (Fig.A2.f, Appendix) is not that clear, we can observe two major components composing the electrostatic graph of *X*_*b*_ (Lorentz time-series). This is a result of the positive and negative values in the structure of the source time-series (*X*_*b*_), illustrating the ability of the electrostatic graph (ESG) algorithm to generate disconnected graphs^1,41,27^. Although connectivity is generally a desirable property in complex networks, the ability of the ESG algorithm to generate disconnected graphs can be insightful for removing past or unnecessary information (noise) of the time-series, therefore proposing avenues for further research. On the other hand, the VGA(*X*_*b*_) graph layout (Fig.A2.f, Appendix) better illustrates a chaotic structure than its spy plot (Fig.A2.b,d Appendix) does, which is more illustrative to a periodic than to chaotic structure.

Next, in Fig.A3 (Appendix) the ESG(*X*_*c*_) spy plot (Fig.A3.a,c, Appendix) configures a tie (along the main diagonal), with an almost constant width, which complies with the stationary structure of the source time-series (*X*_*c*_ = DEOK). Some evidence of stationarity can be also observed in the concentrated (solid-like) pattern of the ESG(*X*_*c*_) graph layout (Fig.A3.e, Appendix). On the other hand, neither the VGA(*X*_*c*_) spy plot (Fig.A3.b,d) nor graph layout (Fig.A3.f, Appendix) are illustrative of a stationary structure describing the original time-series (*X*_*c*_).

In Fig.A4 (Appendix), the ESG(*X*_*d*_) spy plot also configures a tie (along the main diagonal) with repeated knot-concentrations (Fig.A4.a,c, Appendix), which complies with the periodic structure of the source time-series (*X*_*d*_ = SUNSPOTS). Some insightful indications of such periodicity can be also observed in the clustered (torus-like) pattern that is shown in the ESG(*X*_*d*_) graph layout (Fig.A4.e, Appendix). On the other hand, the VGA(*X*_*c*_) spy plot (Fig.A4.b,d, Appendix) has an interesting periodic pattern, which is slightly mixed by the square areas of the other connections. However, the VGA(*X*_*d*_) graph layout (Fig.A4.f, Appendix) does not appear illustrative of the periodic structure describing the source time-series (*X*_*d*_).

Finally, the ESG(*X*_*e*_) spy plot configures a tie (along the main diagonal) with repeated slightly thicker segments (Fig.A5.a,c, Appendix), which can relate to the cyclical structure describing the source time-series (*X*_*e*_ = TEMP). However, such cyclical structure is almost hidden in the chain pattern of graph components that have an odd arrangement in the ESG(*X*_*d*_) graph layout (Fig.A5.e, Appendix). Periodicity can become clearer whether the layout will be further stretched to succeed symmetric arrangement similar to this of Fig.A4.e (Appendix). On the other hand, the VGA(*X*_*c*_) spy plot shapes a clearer periodic pattern (Fig.A5.b,d, Appendix), which (although difficult) can be observed in the graph layout (Fig.A5.f, Appendix). Overall, the proposed ESG algorithm appears at least as capable as the VGA is in generating graphs of topologies representative of their source time-series. This observation will be also quantitatively tested in the following sections.

### Correlation analysis

To compare patterns in data variability between the source and the ESG and VGA node-series (see Fig. A6-A10, Appendix), we apply a Pearson’s bivariate correlation analysis, the results of which are shown in Table 2. Amongst the available correlation coefficients, we compare concordant pairs (*r*(*X*,*X*_ESG_(*z*), *r*(*X*,*X*_VGA_(*z*)|*z* = *k*, *C*, *CB*, *CC*, and *CE*) between ESG and VGA node-series and we denote pairwise maxima (max{(*r*(*X*,*X*_ESG_(*z*), *r*(*X*,*X*_VGA_(*z*)}) in bold font. Cases with the *X*_ESG_(*s*) node-series are paired with those of corresponding degree *X*_VGA_(*k*), due to the similarity of the measures of node degree (*k*) and node strength (*s*), for the binary and weighted networks. Within this context, according to Table 2, in the case of the *X*_*a*_ time-series, the variability of the ESG node-series is overall closer to this of the source time-series (*X*_*a*_) than the variability of the VGA node-series overall is, because the ESG node-series count 5 out of 6 maxima, whereas the VGA node-series count just one. This observation implies that the ESG transformation generates graphs that better preserve fluctuations with a linear trend of the original time-series than the VGA does. On the contrary, in the case of the chaotic time-series (*X*_*b*_), the VGA node-series count 5 out of 6 maxima (a double count is given for the *k*,*s* pair), whereas the ESG node-series count just one. This observation implies that the VGA transformation better preserves chaotic fluctuations of the original time-series than the ESG does.

**Table 2:** Results of the Pearson’s bivariate correlation analysis.

**Variable*X***_***i***_(source time-series)	**Measure**	**Variable*X***_***ij***_(*z*^(a)^) (node-series)										
		*j* = VGA					*j* = ESG					
		*z* = *k*	*z* = *C*	*z* = *CB*	*z* = *CC*	*z* = *CE*	*z* = *k*	*z* = *s*^(b)^	*z* = *C*	*z* = *CB*	*z* = *CC*	*z* = *CE*
*i* = *a* (AIR)	*r*(*X*_*i*_,*X*_*ij*_(*z*))	0.331**	-0.158	0.250**	-0.232**	0.254**	0.805**	0.981**	0.358**	-0.144	-0.359**	0.837**
	sig.^(c)^	0.000	0.059	**0.002**	0.005	0.002	**0.000**	**0.000**	**0.000**	0.085	**0.000**	**0.000**
	*n*^(d)^						144					
*i* = *b* (CHAOS)	*r*(*X*_*i*_,*X*_*ij*_(*z*))	0.516**	0.019	0.188**	-0.185**	0.201**	-0.008	0.002	**-0.022**	0.020	0.103**	-0.132**
	sig	**0.000**	0.401	**0.000**	**0.000**	**0.000**	0.712	0.919	**0.345**	0.375	0.000	0.000
	*n*						1900					
*i* = *c* (DEOK)	*r*(*X*_*i*_,*X*_*ij*_(*z*))	0.354**	-0.570**	0.148**	-0.140**	0.157**	0.890**	0.989**	-0.549**	0.179**	0.088**	0.725**
	sig	0.000	**0.000**	0.000	**0.000**	0.000	**0.000**	**0.000**	0.000	**0.000**	0.000	**0.000**
	n						5000					
*i* = *d* (SUNSPOTS)	*r*(*X*_*i*_,*X*_*ij*_(*z*))	0.496**	-0.666**	0.478**	-0.567**	0.309**	0.768**	0.773**	.^b^	0.593**	0.712**	0.733**
	sig	0.000	**0.000**	0.000	0.000	0.000	**0.000**	**0.000**		**0.000**	**0.000**	**0.000**
	*n*						280					
*i* = *e* (TEMP)	*r*(*X*_*i*_,*X*_*ij*_(*z*))	0.437**	-0.439**	0.211**	-0.520**	0.164**	0.908**	0.944**	0.405**	0.123**	-0.133**	0.752**
	sig	0.000	**0.000**	**0.000**	**0.000**	0.000	**0.000**	**0.000**	0.000	0.000	0.000	**0.000**
	*n*						3650					

^a^*k* = node degree, *C* = clustering coefficient, *CC* = closeness centrality, *CB* = betweenness centrality, *CE* = eigenvector centrality.

c. In pairwise consideration, *X*_ESG_(*s*) is paired with the *X*_*VGA*_(_*k*_).

c. 2-tailed significance.

d. Number of cases.

**Correlation is significant at the 0.01 level (2-tailed).

Cases shown in **bold** indicate maximum coefficients (in absolute terms) between concordant ESG versus VGA pairs, max{*r*(*X*,*X*_ESG_(*z*), *r*(*X*,*X*_VGA_(*z*)}.

Underlined cases indicate max coefficients (in absolute terms) within each row (for each time-series type).

In the case of the *X*_*c*_ (DEOK), the ESG node-series count 4 out of 6 maxima, whereas the VGA node-series count 2 out of 6, which implies that the ESG transformation better preserves stationary fluctuations of the original time-series than the ESG does. In the case of the *X*_*d*_ (SUNSPOTS), the ESG node series count 5 out of 6 maxima, whereas the VGA node-series count just one, which implies that the ESG transformation better preserves periodical fluctuations of the original time-series than the VGA does. In the case of the *X*_*e*_ (TEMP) both the ESG and the VGA node-series count 3 out of 6 maxima, showing a balanced performance. As far as the measure of strength (*s*) (see Fig. A11, Appendix) is concerned, the analysis shows that, for all types of time-series except the chaotic one (*X*_*b*_, CHAOS), the ESG node-series have higher performance than the VGAs. Overall, this pair-wise consideration illustrates that the variability of ESG node-series is closer to the source time-series (*X*_*i*_) than of the VGAs, since the first count 18 out of 30 maximum cases, whereas the latter count 12 out of 30 maxima.

### Test of the linear trend

The test of the linear trend was applied to ESG and VGA node-series associated with the *X*_*a*_ (AIR) time-series, which is a time-series with a known linear trend. The results of the analysis are shown in Table 3, where first it can be observed that the source (*X*_*a*_: AIR) time-series is well described by a linear regression model (*R*^2^ = 0.8536). However, none of the VGA node-series can sufficiently retain this linear structure, as is evident by the low coefficients of determination ranging from *R*^2^ = 0.0002 to *R*^2^ = 0.0132.

**Table 3:** Linear regression fittings for the *X*_*a*_ (Air) time-series.

Time-series/ Measure		Linear regression	
		Mathematical expression	Determination
***x***_***a***_ (AIR)		*y* = 2.6572*x* + 87.653	***R***^**2**^** = 0.8536**
VGAs	***k***(***x***_*a*_)	*y* = 0.0118*x* + 7.0629	*R*^2^ = 0.0096
	***C***(***x***_*a*_)	*y* = 0.0006*x* + 0.7174	*R*^2^ = 0.0132
	***CB***(***x***_*a*_)	*y* = 0.174*x* + 141.63	*R*^2^ = 0.0003
	***CC***(***x***_*a*_)	*y* = -0.0002*x* + 3.1682	*R*^2^ = 0.0002
	***CE***(***x***_*a*_)	*y* = 0.0001*x* + 0.2013	*R*^2^ = 0.0009
ESGs	***k***(***x***_*a*_)	*y* = 0.2149*x* + 13.656	***R***^**2**^** = 0.6916**
	***s***(***x***_*a*_)	*y* = 4813.4*x* – 62,583	***R***^**2**^** = 0.8012**
	***C***(***x***_*a*_)	*y* = 0.0009*x* + 0.6969	*R*^2^ = 0.1985
	***CB***(***x***_*a*_)	*y* = -1.5134*x* + 315.61	*R*^2^ = 0.0663
	***CC***(***x***_*a*_)	*y* = -0.0106*x* + 4.6484	*R*^2^ = 0.2034
	***CE***	*y* = 0.0069*x*—0.1143	***R***^**2**^** = 0.7579**

Cases shown in **bold** indicate high (> 0.65) coefficients of determination.

Underlined cases highlight the max coefficient, in total.

On the contrary, the ESG node-series of degree *X*_ESG_(*k*), strength *X*_ESG_(s), and eigenvector centrality *X*_ESG_(CE) have a considerable linear structure, as is denoted by their respective coefficients of determination *R*^2^ = 0.6916, *R*^2^ = 0.8012, and *R*^2^ = 0.7579. It should be noted that among these cases, the strength node-series *X*_ESG_(s) have the highest determination. Overall, this analysis illustrates that the ESG algorithm appears more capable than the VGA in generating graphs that can preserve aspects of the linear trend of the source time-series.

### Detection of chaotic structure

In this part of the analysis, the correlation versus the embedding dimension diagrams (*v*,*m*) of the VGA and the ESGs node-series are compared for preserving the chaotic structure of the source time-series *X*_*b*_ (CHAOS), which is already known as a chaotic time-series constructed on the Lorenz equations. The results are shown in Fig. A7 (Appendix), where all (*v*,*m*) diagrams of the ESG node-series (except this of eigenvector centrality *X*_*b*,ESG_(*CE*)) illustrate the chaotic structure, but of different characteristics than the source chaotic time-series *X*_*b*_. However, the (*v*,*m*) diagrams of strength *X*_*b*,ESG_(*s*) and the original time-series *X*_*b*_ almost coincide, a fact that implies a relevant chaotic structure between these time-series. On the other hand, the degree *X*_*b*,VGA_(*k*), and possibly the eigenvector centrality *X*_*b*,VGA_(*CE*) VGA node-series illustrate a chaotic structure of high dimensionality, which are also of different characteristics than the original chaotic time-series *X*_*b*_. Overall, the chaos analysis shows that the ESG is a more capable transformation in incorporating the chaotic structure of the source time-series in the network topology. Particularly, the measure of strength shows the most relevant chaotic structure that almost coincides with this of source time-series.

### Detection of stationarity

The test of stationarity was applied to the *X*_*c*_ (DEOK) time-series, which is a part of an already known stationary time-series. The results of the analysis are shown in Table 4, where, first, it can be observed that is 7.03% likely for *X*_*c*_ to have a unit-root and thus to be a non-stationary time-series. This result implies that the null-hypothesis (stating a null unit-root) cannot be rejected, and thus that the source (*X*_*c*_) time-series cannot be considered as a stationary one. As it can be observed, the results for all VGA node-series imply that all cases are statistically safe to be considered as stationary series, which opposes the indication of the original time-series.

**Table 4:** ADF test for stationarity of the *X*_*c*_ (DEOK) time-series.

		ADF test			
		*h*^(a)^	*p*-Value	DFt (stat)	cValue
***x***_**c**_ (DEOK)		**0**	**0.0703**	**-1.7876**	**-1.9416**
VGAs	***k***	1	**0.0010**^**(b)**^	-16.8555	**-1.9416**
	***C***	1	**0.0010**	-8.8160	
	***CB***	1	**0.0010**	-63.9394	
	***CC***	1	**0.0176**	**-2.3683**	
	***CE***	1	**0.0010**	-28.4443	
ESGs	***k***	**0**	0.2176	**-1.1860**	**-1.9416**
	***s***	**0**	0.3876	**-0.7177**	
	***C***	**0**	0.3458	**-0.8359**	
	***CB***	1	0.0010	**-20.2674**	
	***CC***	**0**	0.1739	**-1.3179**	
	***CE***	**0**	0.2251	**-1.1656**	

^a^*h* = 0 indicates failure to reject the unit-root null (indication: non-stationary series).

*h* = 1 indicates rejection of the unit-root null in favor of the alternative model (indication: stationary series).

b. Cases shown in **bold** are the closest between concordant measures to the source time-series scores.

On the other hand, the ESG results imply that 4 out of 5 ESG node-series cannot be considered as stationary ones and thus resembling the structure of the original time-series. An interesting observation here is that the *p*-values of the VGA node-series are (although insufficient indications to retain the null hypothesis) closer than those of the ESG node-series, in terms of distance. These results imply that the non-stationary effects, which are immanent in the source time-series, probably appear more intensely in the structure of the ESG node-series than of the VGA ones.

### Detection of periodicity and cyclical structure

This part of analysis builds on bivariate correlations, which are applied to autocorrelation variables *ACF*(*X*) that are defined in relation (14) with lag 1,2,…,30, where *X* = *X*_*d*_ (SUNSPOTS time-series), *X*_*e*_ (TEMP time-series), *k* (degree node-series), *C* (clustering coefficient node-series), *CB* (betweenness centrality node-series), *CC* (closeness centrality node-series), and *CE* (eigenvector centrality node-series). The results of the analysis are shown in Table 5, where the correlation coefficients *r*_*XY*_ and their significances are provided, with *X*$\in${ *ACF*(*X*_*d*_), *ACF*(*X*_*e*_)} and *Y*$\in${*ACF*_*i*_(*k*), *ACF*_*i*_(*s*), *ACF*_*i*_(*C*), *ACF*_*i*_(*CB*), *ACF*_*i*_(*CC*), *ACF*_*i*_(*CE*) | where *i* = *VGA*, *ESG*}.

**Table 5:** Correlations of ACFs^(*)^ between the source and the node-series (Sunspots and Temp).

***Y*-variable**		***X-*variable**			
		*ACF*(*X*_*d*_) [SUNSPOTS]		*ACF*(*X*_*e*_) [TEMP]	
		*r*_*XY*_	Sig	*r*_*XY*_	Sig
VGA	*ACF*(*k*)	**0.8397**	0	-0.0803	0.613
	*ACF*(*C*)	0.7953	0	-0.1311	0.408
	*ACF*(*CB*)	-0.082	0.6057	0.2807	0.0717
	*ACF*(*CC*)	0.4225	0.0053	**1**	0
	*ACF*(*CE*)	**0.823**	0	0.3306	0.0325
ESG	*ACF*(*k*)	0.6513	0	**0.9999**	0
	*ACF*(*s*)	**0.9379**	0	**0.9196**	0
	*ACF*(*C*)	NaN**	NaN	**0.9999**	0
	*ACF*(*CB*)	0.2398	0.1261	**0.9998**	0
	*ACF*(*CC*)	**0.7105**	0	0.9999	0
	*ACF*(*CE*)	0.5601	0.0001	**0.9999**	0

*ACFs are computed on lags from 1:30 (relation 13).

**Not a number (unable to compute due to zero entries).

Cases shown in **bold** indicate max coefficients (in absolute terms) according to pairwise (ESG *vs*. VGA) comparisons (only statistically significant cases are shown).

Underlined cases indicate max coefficients (in absolute terms) within each column (for each time-series type).

For the case of *X*_*d*_ (SUNSPOTS) time-series, we can observe that 4 out of 6 VGA node-series (*k*, *k≡s*, *C*, *CE*) and 3 out of 6 ESG node-series (*k*, *s*, *CC*) are significantly correlated with the original time series *X*_*d*_. Amongst these significant results, the VGA node-series have 2 maxima of concordant pairs, whereas the ESG node-series have also 2 maxima. Moreover, the node-series of strength (*X*_*d,*ESG_(*s*)) has the highest correlation coefficient amongst all available node-series for the SUNSPOTS (*X*_*d*_) typology, illustrating a better performance of the ESG algorithm to preserve periodicity, probably due to its capability in generating weighted electrostatic networks. For the case of *X*_*e*_ (TEMP) time-series, 1 VGA node-series (closeness centrality) is significantly correlated with the source time-series, where all ESG node-series are significantly correlated with the original time-series. In terms of pairwise comparisons, the VGA node-series count 1 (out of 6) maximum case, whereas the ESG node-series count 5 out of 6 maxima. However, although is high, the strength does suggest the highest of the maxima of the TEMP (*X*_*e*_) time-series concordant pairs. Overall, this analysis shows that the ESG node-series appear more capable than the VGA ones in preserving periodic and cyclical characteristics of the source time-series.

## Conclusions

This paper proposed a new algorithm, the Electrostatic Graph Algorithm (ESG), for converting a time-series into a graph (complex network). The ESG builds on the conceptualization of considering a time-series as a series of stationary and electrically charged particles, on which Coulomb-like forces can be computed. The proposed algorithm provides an added value to complex network analysis of time-series due to its ability to produce weighted graphs, which is currently not applicable. This additional property was quantitatively examined in this paper and was found to produce graphs that are more representative of the structure of the source (original) time-series, implying that the proposed algorithm suggests a transformation that is more natural rather than algebraic, in comparison with the existing methods. In particular, the analysis showed that the ESG node-series can better preserve the linear trend and stationary structural properties of the source time-series in comparison with the VGA node-series and that they appear slightly better in preserving periodical and cyclical structural properties of the original time-series than the VGA node-series can. On the other hand, the VGA node-series appeared slightly better in preserving the chaotic structural properties of the original time-series in comparison with the ESG node-series, which complies with the claim of the VGA authors regarding the added value of their algorithm. However, in almost all the parts of the analysis, the ESG node-series of the measure of strength outperformed their concordant VGA node-series. This result highlighted the added value of the proposed algorithm in generating weighted graphs, in which the measure of node strength can only be computed. Therefore the ESG algorithm attributes to the generated graphs information that is more representative of the source time-series, due to the weights included in the graph structure. Another property of the proposed ESG algorithm to generate disconnected graphs was indirectly examined by the detection of chaotic and periodic structures, where the ESG algorithm sufficed to provide disconnected graphs, whereas the VGA did not. This analysis showed that insufficient connectivity does not restrict the ESG node-series to preserve the structural characteristics of the source time-series, since the generated electrostatic graphs were representative of the structure of the original time-series. The authors believe that the property of insufficient connectivity introduces avenues of further research in the field of noise reduction in the time-series analysis. Other avenues of further research can emerge towards the direction of either choosing the optimum or most representative connectivity threshold to produce the ESGs or examining the applicability of the proposed algorithm to solve problems where standard methods fail to analyze efficiently the time-series, such as the time evolution of stock price, within the framework of Black Scholes model, and others. The overall approach also suggests a methodological framework for evaluating the structural relevance between the source time-series and their associated graphs produced by any possible transformation.

## Supplementary Information

Supplementary Information.
