# Supplementary material for: An electrostatics method for converting a time-series into a weighted complex network
Source: Sci Rep. 2021 Jun 3;11:11785. doi: 10.1038/s41598-021-89552-2 (PMC8175385; doi:10.1038/s41598-021-89552-2)
Supplement: Supplementary file 1 — Supplementary Information. [file 41598_2021_89552_MOESM1_ESM.docx]

**An electrostatics method for converting a time-series into a weighted complex network**

**Dimitrios Tsiotas^1,2,3^, Lykourgos Magafas^3^, and Panos Argyrakis^3,4^**

^1^. Department of Regional and Economic Development, Agricultural University of Athens, Amfissa, Greece.

^2^. Adjunct Academic Staff, School of Social Sciences, Hellenic Open University, Athens 10677, Greece.

^3^. Laboratory of Complex Systems, Department of Physics, International Hellenic University, Kavala, Greece.

^4^. Department of Physics, Aristotle University of Thessaloniki, Thessaloniki, Greece.

**^*^***Correspondence*: tsiotas@aua.gr.

**Abstract**

This paper proposes a new method for converting a time-series into a weighted graph (complex network), which builds on electrostatics in physics. The proposed method conceptualizes a time-series as a series of stationary, electrically charged particles, on which Coulomb-like forces can be computed. This allows generating electrostatic-like graphs associated with time-series that, additionally to the existing transformations, can be also weighted and sometimes disconnected. Within this context, this paper examines the structural similarity between five different types of time-series and their associated graphs that are generated by the proposed algorithm and the visibility graph, which is currently the most popular algorithm in the literature. The analysis compares the source (original) time-series with the node-series generated by network measures (that are arranged into the node-ordering of the source time-series), in terms of a linear trend, chaotic behaviour, stationarity, periodicity, and cyclical structure. It is shown that the proposed electrostatic graph algorithm generates graphs with node-measures that are more representative of the structure of the source time-series than the visibility graph. This makes the proposed algorithm more natural rather than algebraic, in comparison with existing physics-defined methods. The overall approach also suggests a methodological framework for evaluating the structural relevance between the source time-series and their associated graphs produced by any possible transformation.

**Keywords** natural transformation; visibility algorithm; complex network analysis of time-series; pattern recognition.

**APPENDIX**

**
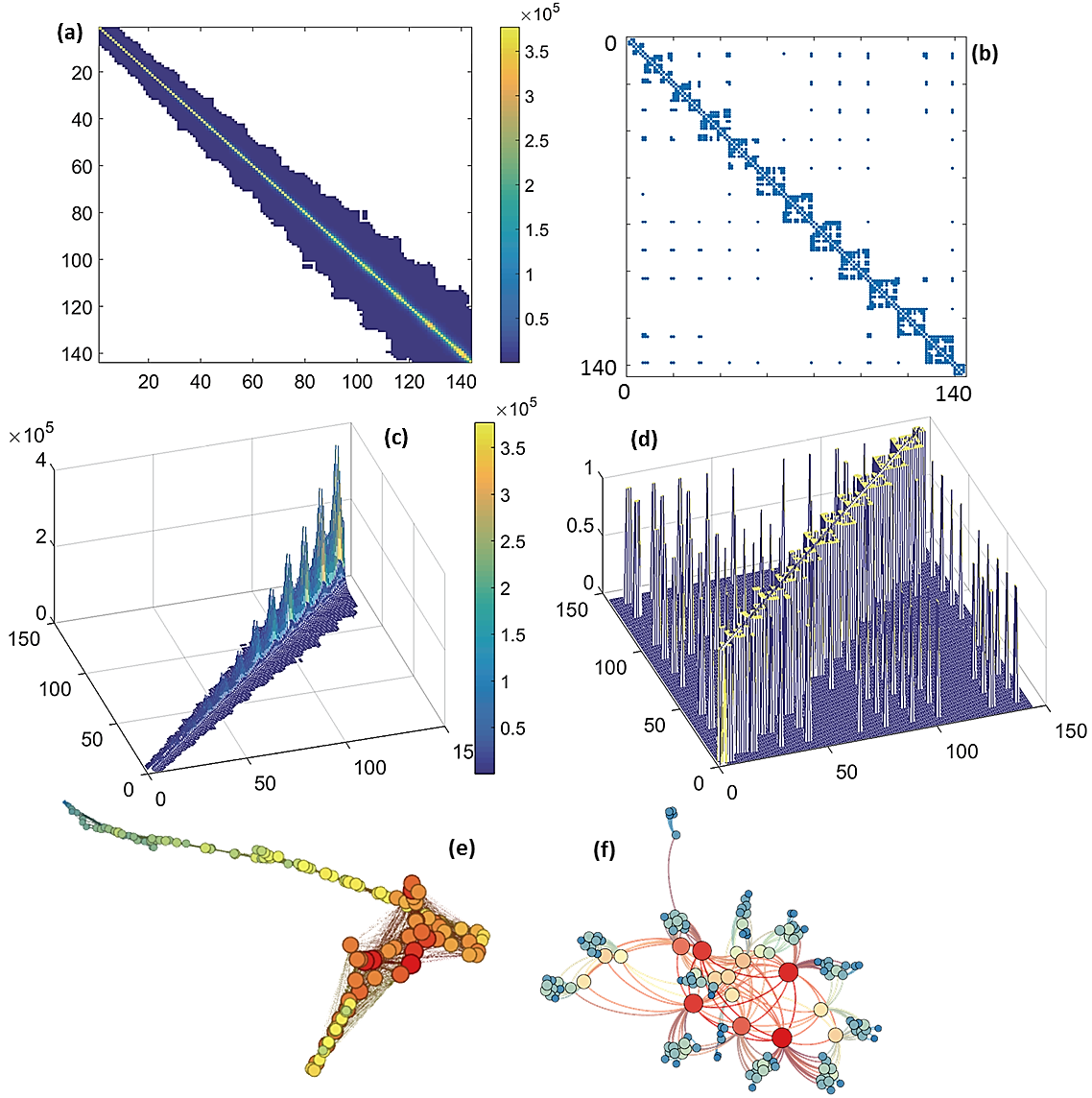
**

**Fig.A1.** (a) Spy plot (weighted) of the ESG connectivity matrix, (b) Spy plot of the NVG adjacency matrix, (c) 3d mesh plot of the ESG connectivity matrix, (d) 3d mesh plot of the NVG adjacency matrix, (e) Force-atlas (Bastian et al. 2009) layout of the ESG, and (f) Force-atlas layout of the NVG. Both ESG and NVG are computed on the air-passengers (*X_a_*) time-series (Fig.5a).


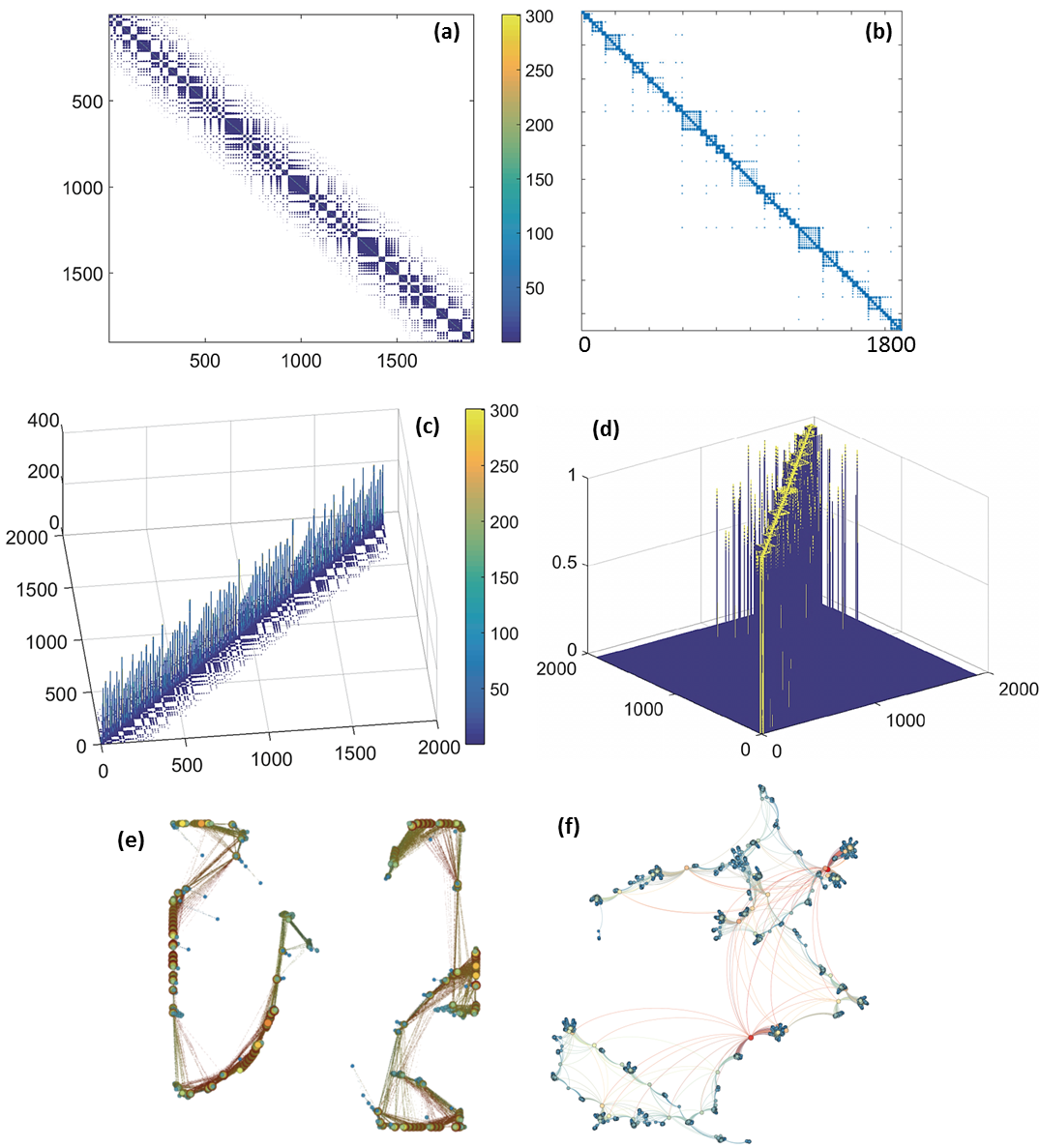


**Fig.A2.** (a) Spy plot (weighted) of the ESG adjacency matrix, (b) Spy plot of the NVG adjacency matrix, (c) 3d mesh plot of the ESG connectivity matrix, (d) 3d mesh plot of the NVG adjacency matrix, (e) Force-atlas (Bastian et al. 2009) layout of the ESG, and (f) Force-atlas layout of the NVG. Both ESG and NVG are computed on the Chaos (*X_b_*) time-series (Fig.5b).


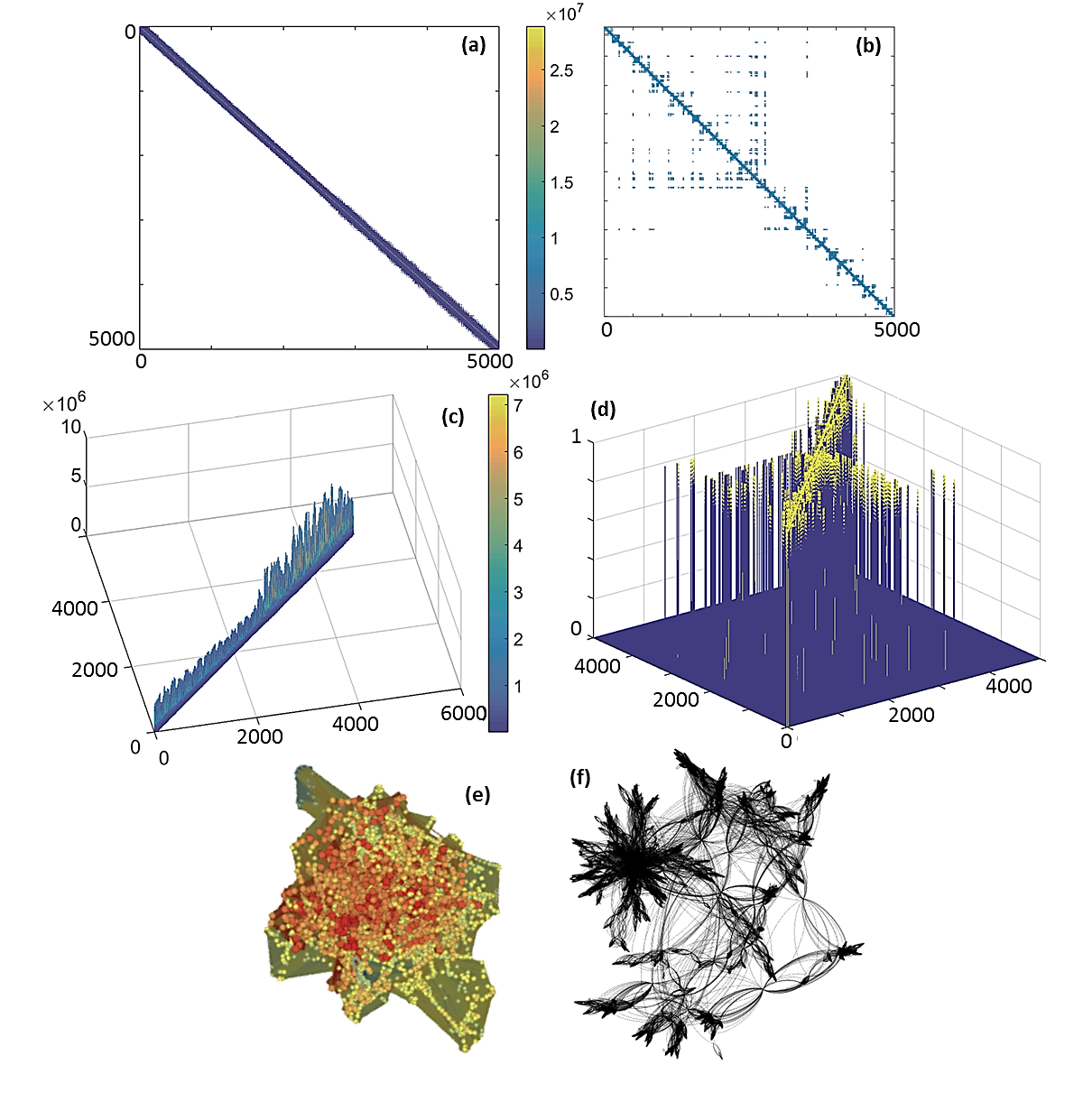


**Fig.A3.** (a) Spy plot (weighted) of the ESG adjacency matrix, (b) Spy plot of the NVG adjacency matrix, (c) 3d mesh plot of the ESG connectivity matrix, (d) 3d mesh plot of the NVG adjacency matrix, (e) Force-atlas (Bastian et al. 2009) layout of the ESG, and (e) Force-atlas layout of the NVG. Both ESG and NVG are computed on the DEOK (*X_c_*) time-series (Fig.5c).


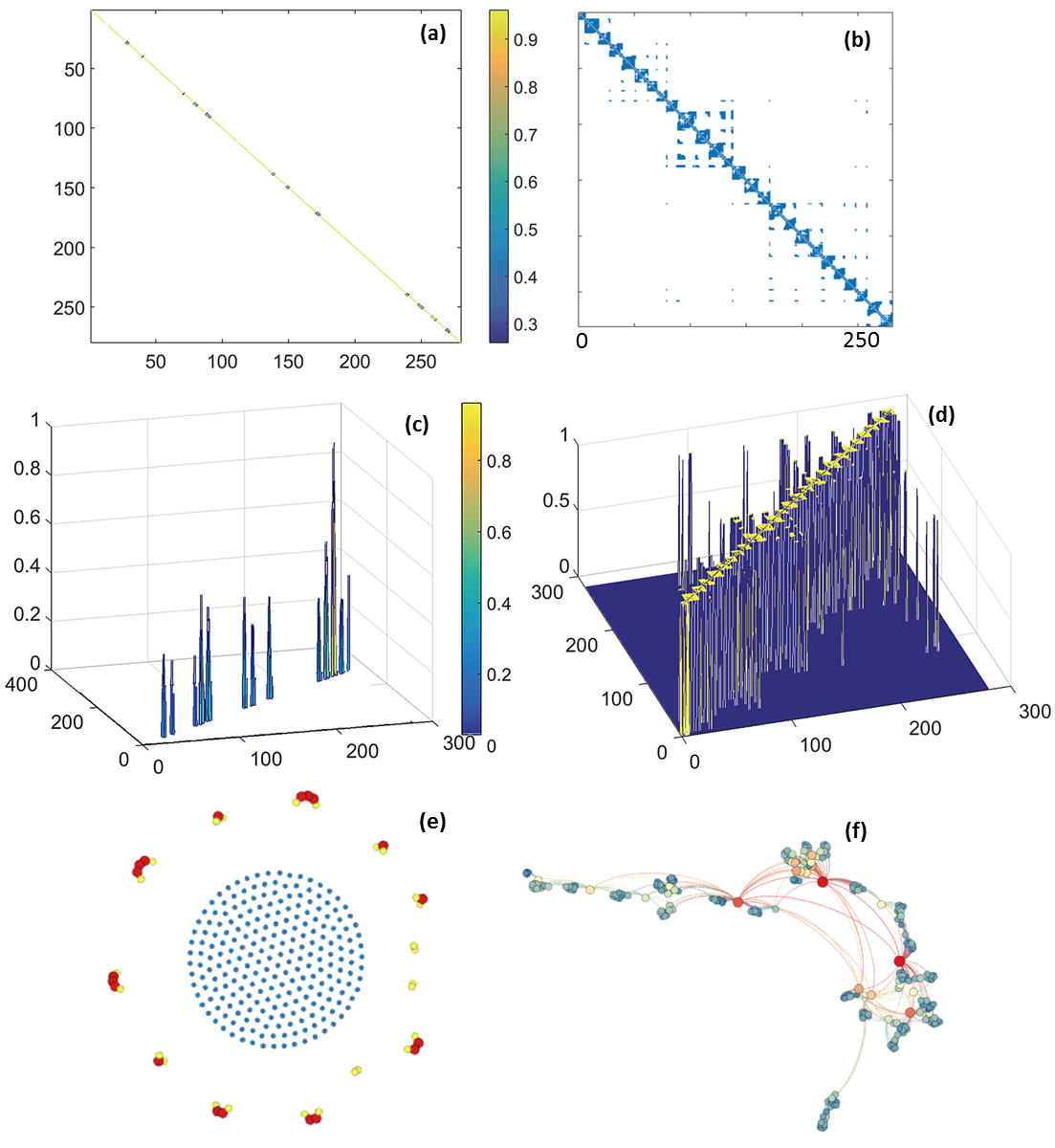


**Fig.A4.** (a) Spy plot (weighted) of the ESG adjacency matrix, (b) Spy plot of the NVG adjacency matrix, (c) 3d mesh plot of the ESG connectivity matrix, (d) 3d mesh plot of the NVG adjacency matrix, (e) Force-atlas (Bastian et al. 2009) layout of the ESG, and (f) Force-atlas layout of the NVG. Both ESG and NVG are computed on the sunspots (*X_d_*) time-series (Fig.5d).


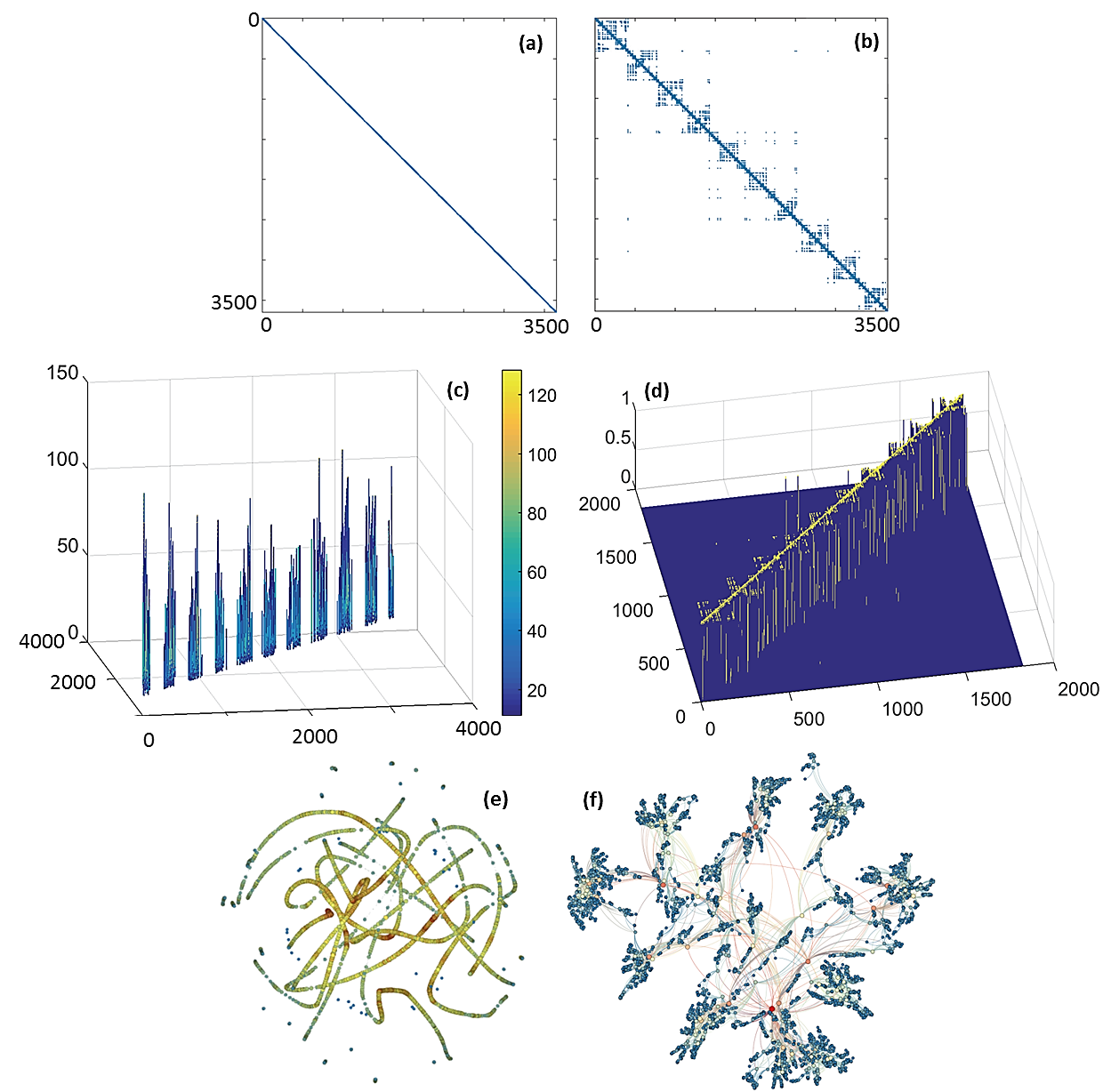


**Fig.A5.** (a) Spy plot (weighted) of the ESG adjacency matrix, (b) Spy plot of the NVG adjacency matrix, (c) 3d mesh plot of the ESG connectivity matrix, (d) 3d mesh plot of the NVG adjacency matrix, (e) Force-atlas (Bastian et al. 2009) layout of the ESG, and (f) Force-atlas layout of the NVG. Both ESG and NVG are computed on the temperature (*X_e_*) time-series (Fig.5e).

**
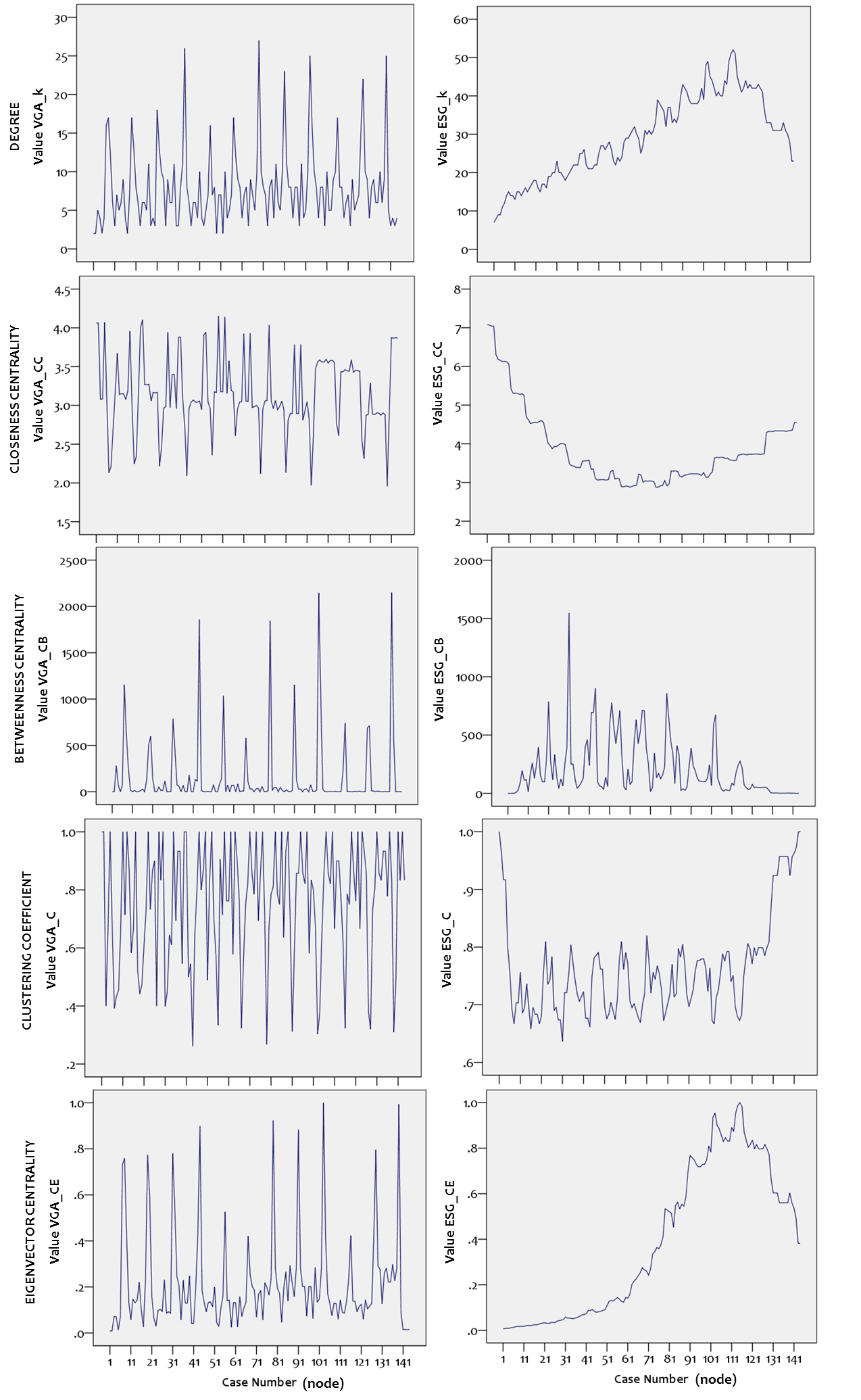
**

**Fig.A6.** Line plots of VGA and ESG node-series for the air (*X_a_*) time-series.

**
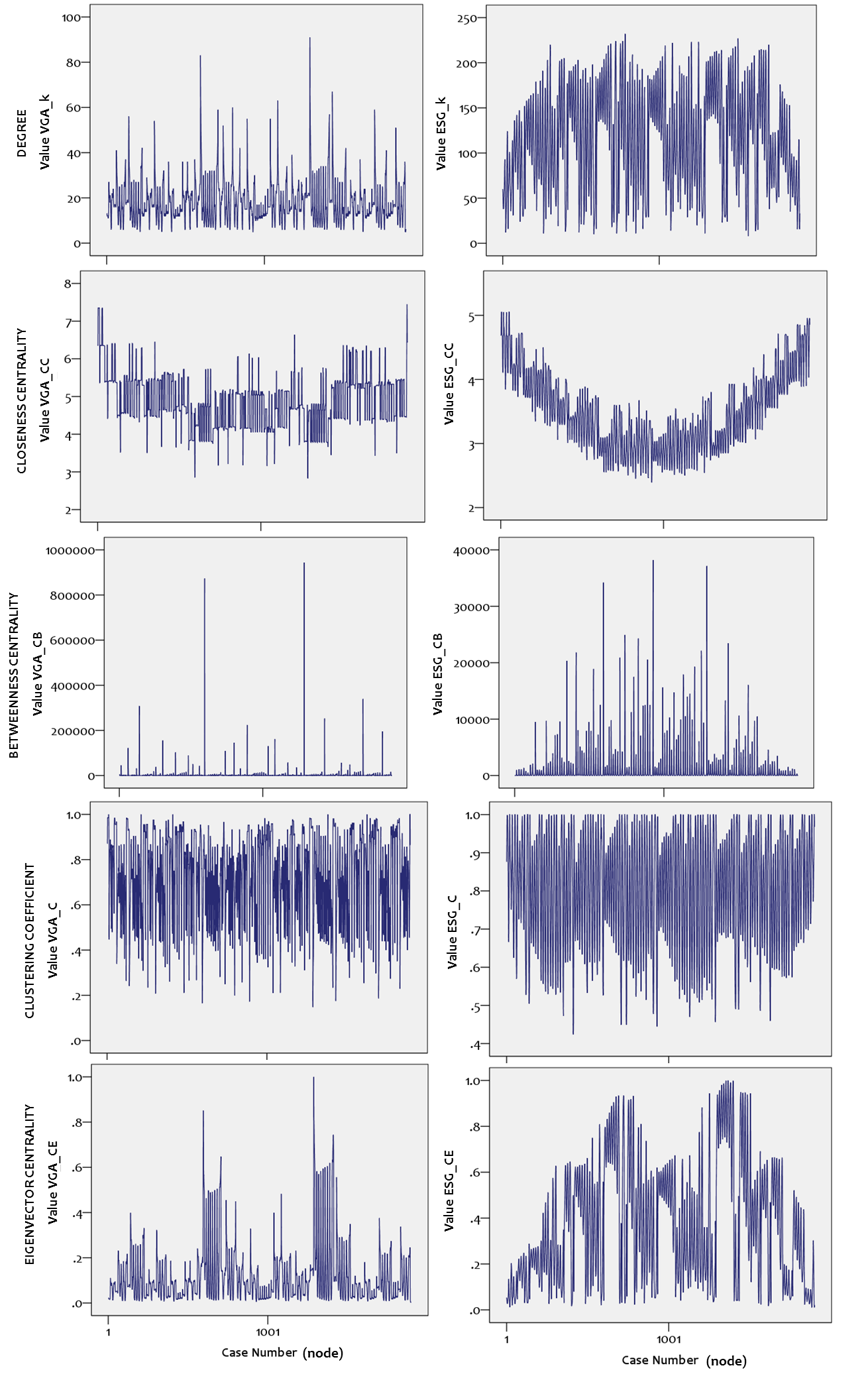
**

**Fig.A7.** Line plots of VGA and ESG node-series for the chaos (*X_b_*) time-series.

**
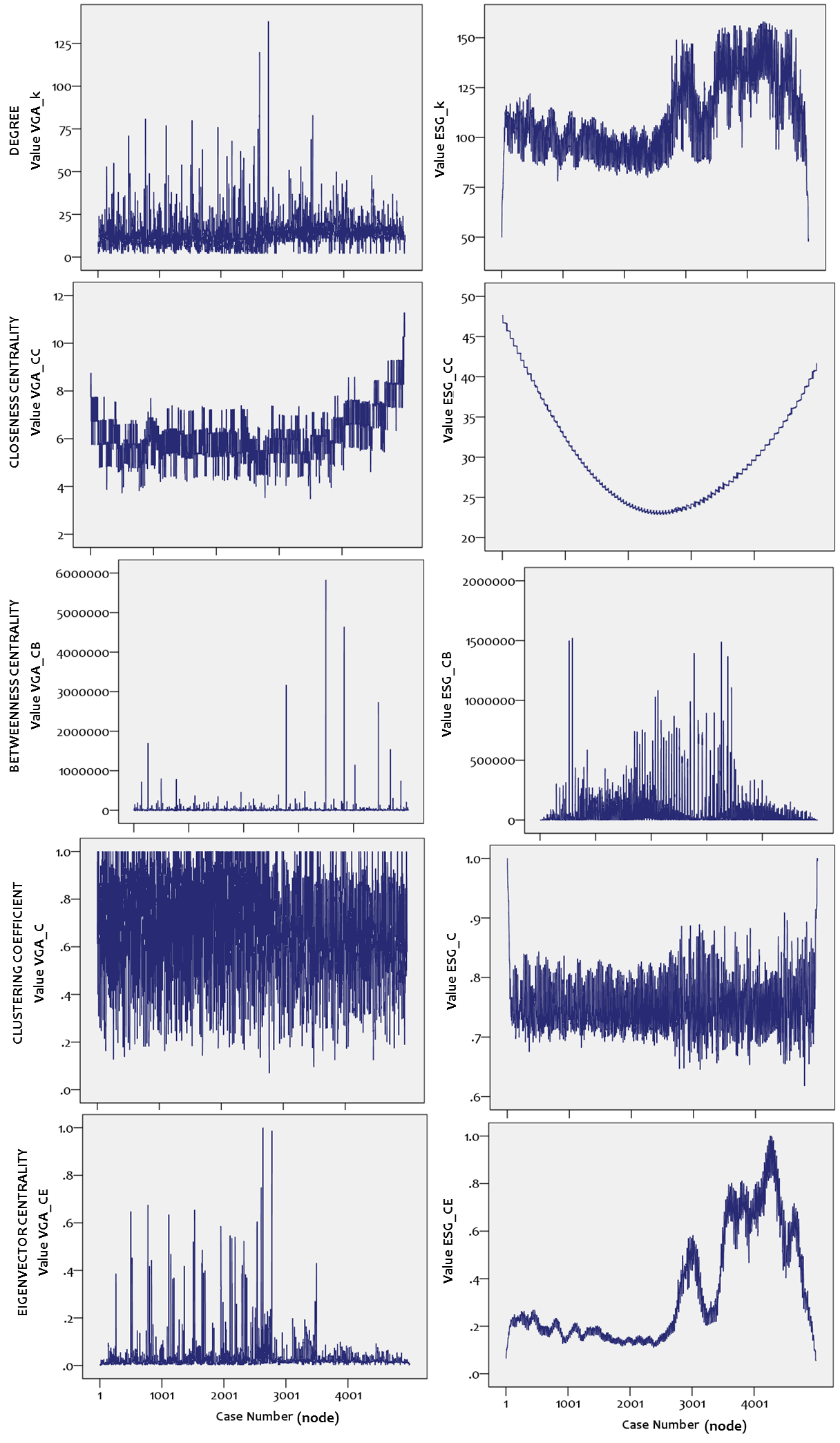
**

**Fig.A8.** Line plots of VGA and ESG node-series for the DEOK (*X_c_*) time-series.

**
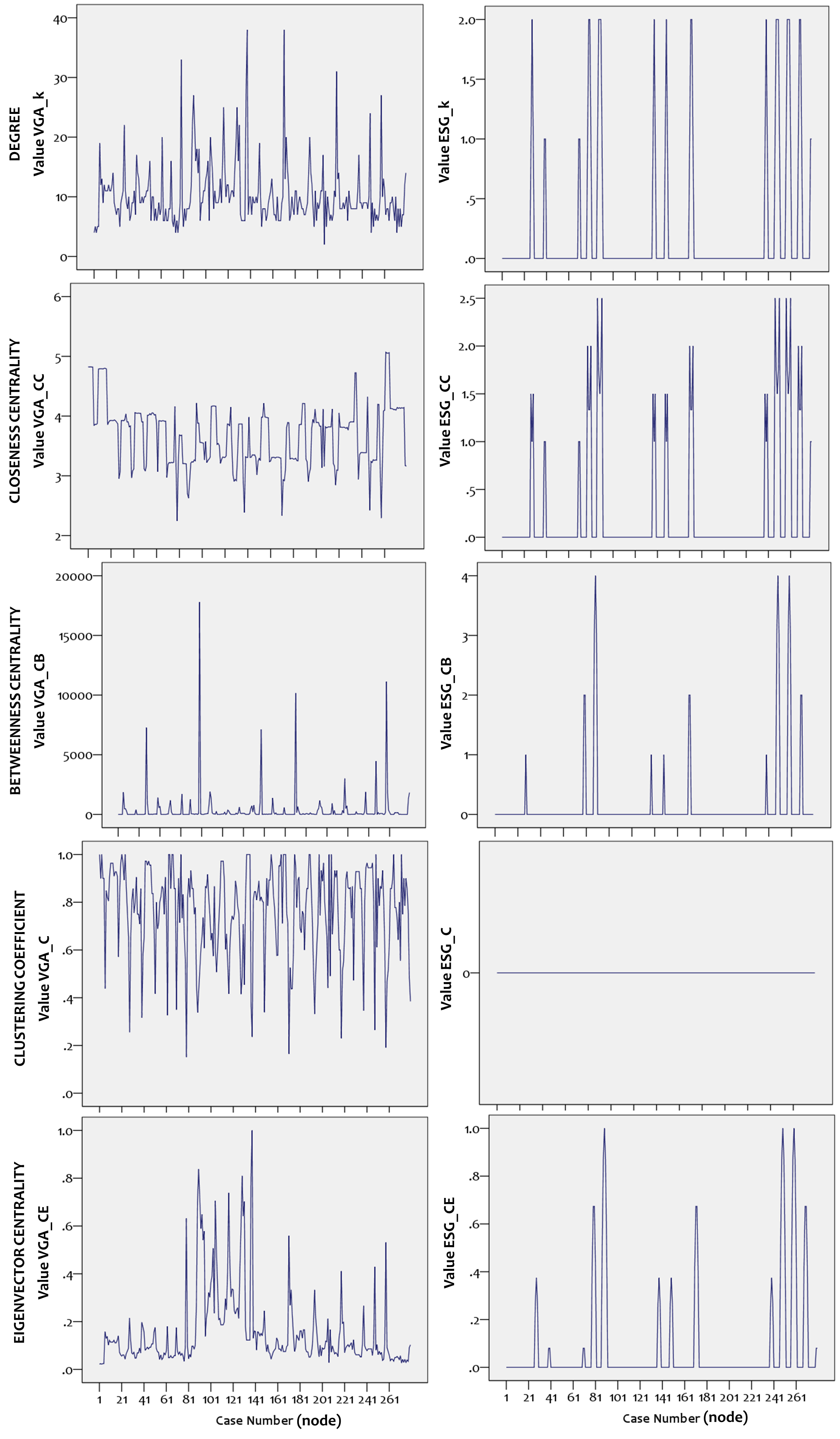
**

**Fig.A9.** Line plots of VGA and ESG node-series for the sunspots (*X_d_*) time-series.

**
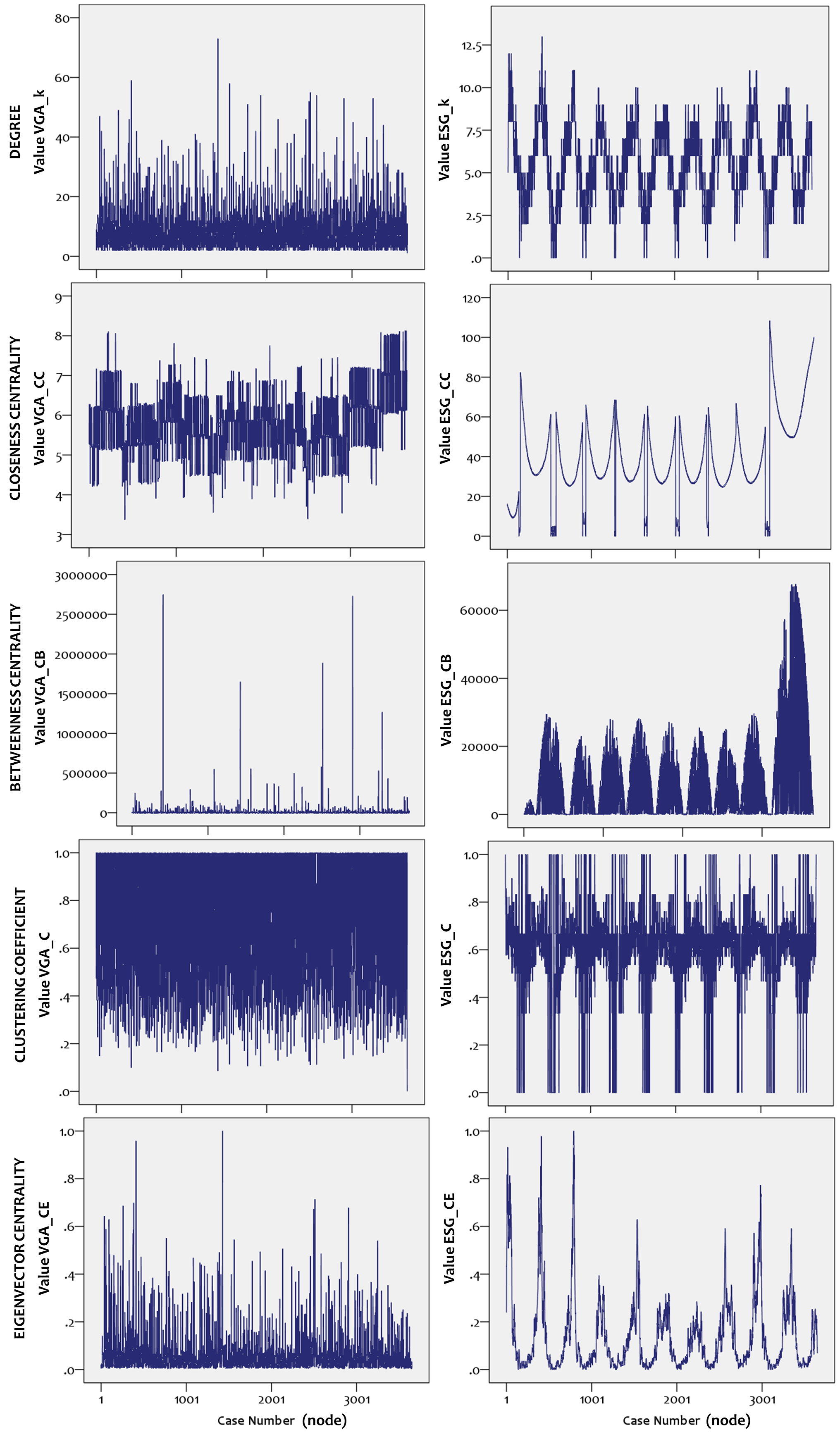
**

**Fig.A10.** Line plots of VGA and ESG node-series for the temp (*X_e_*) time-series.

**
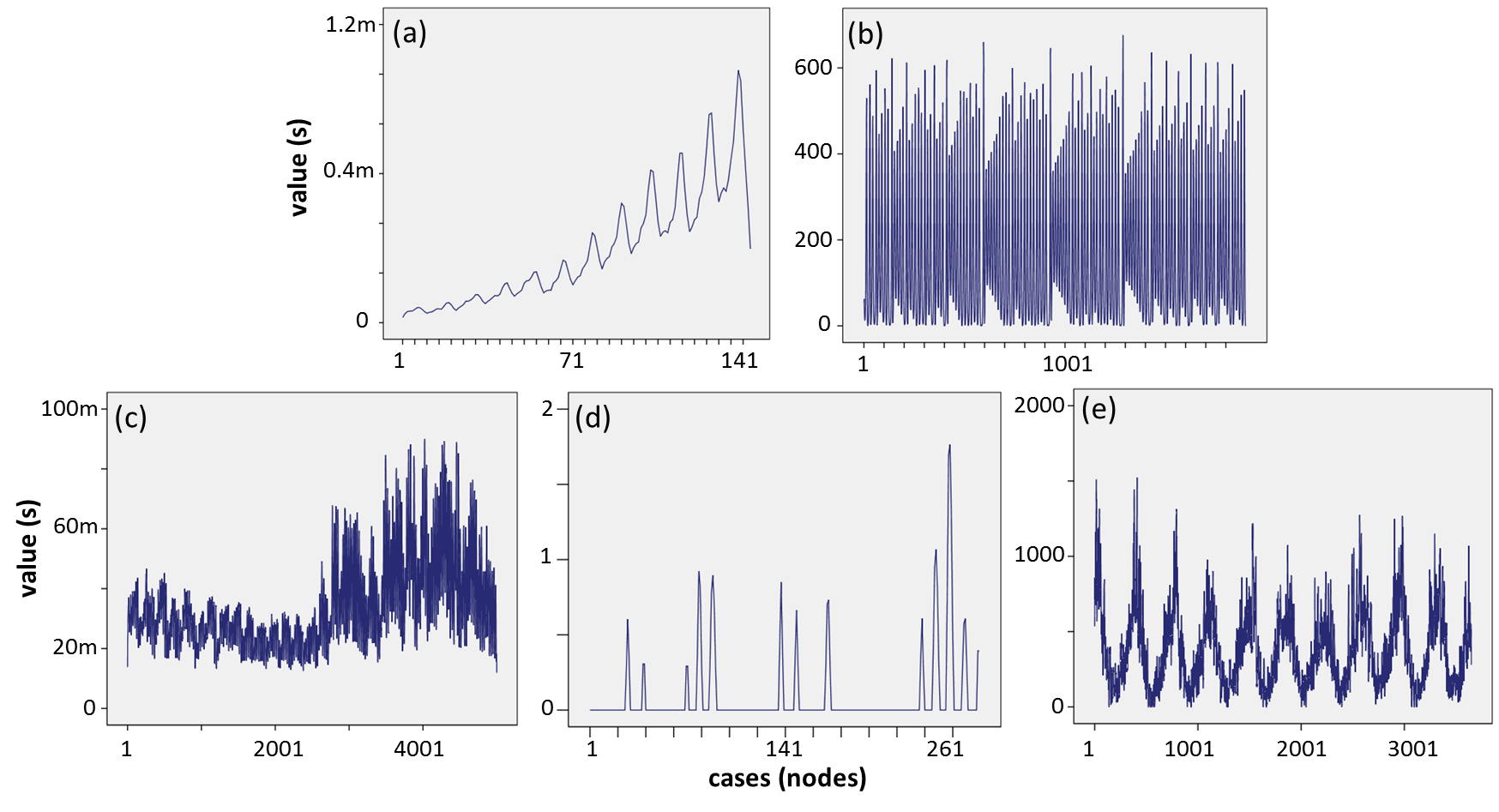
**

**Fig.A11.** The ESG node-series of the measure of strength (*s*), for the available (a) air-passengers (*X_a_*), (b) typical Lorentz chaotic (*X_b_*), (c) DEOK (*X_c_*), (d) periodical (*X_d_*), and (e) cyclic (*X_e_*) source time-series considered in the analysis.

**
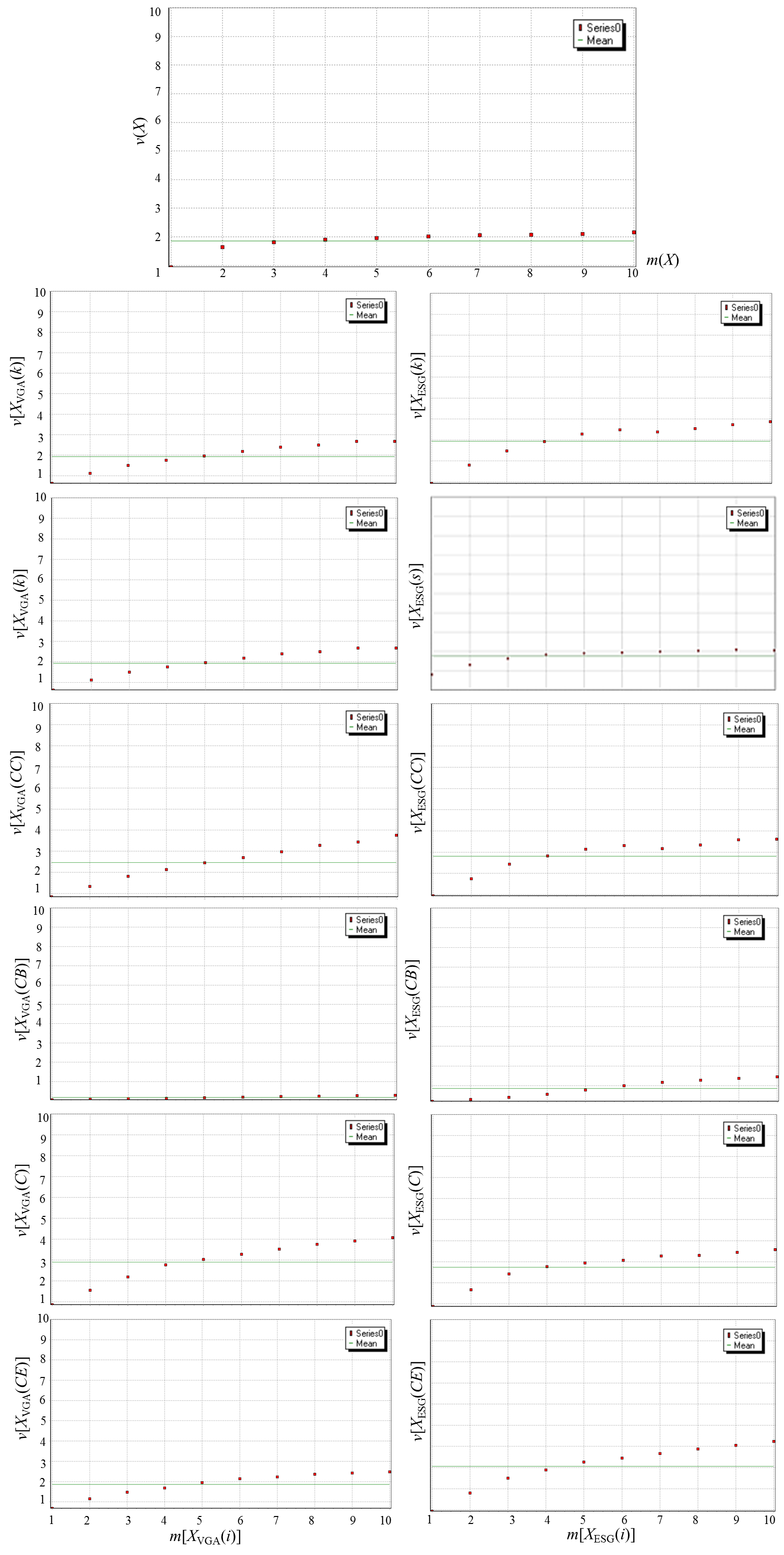
**

**Fig.A12 (part A: degree).** Correlation (*v*) *vs*. embedding dimension (*m*) diagrams (*v*,*m*), of the original (typical Lorentz chaotic) time-series *X_b_* and the VGA and ESGs node-series of degree (*k*).

**
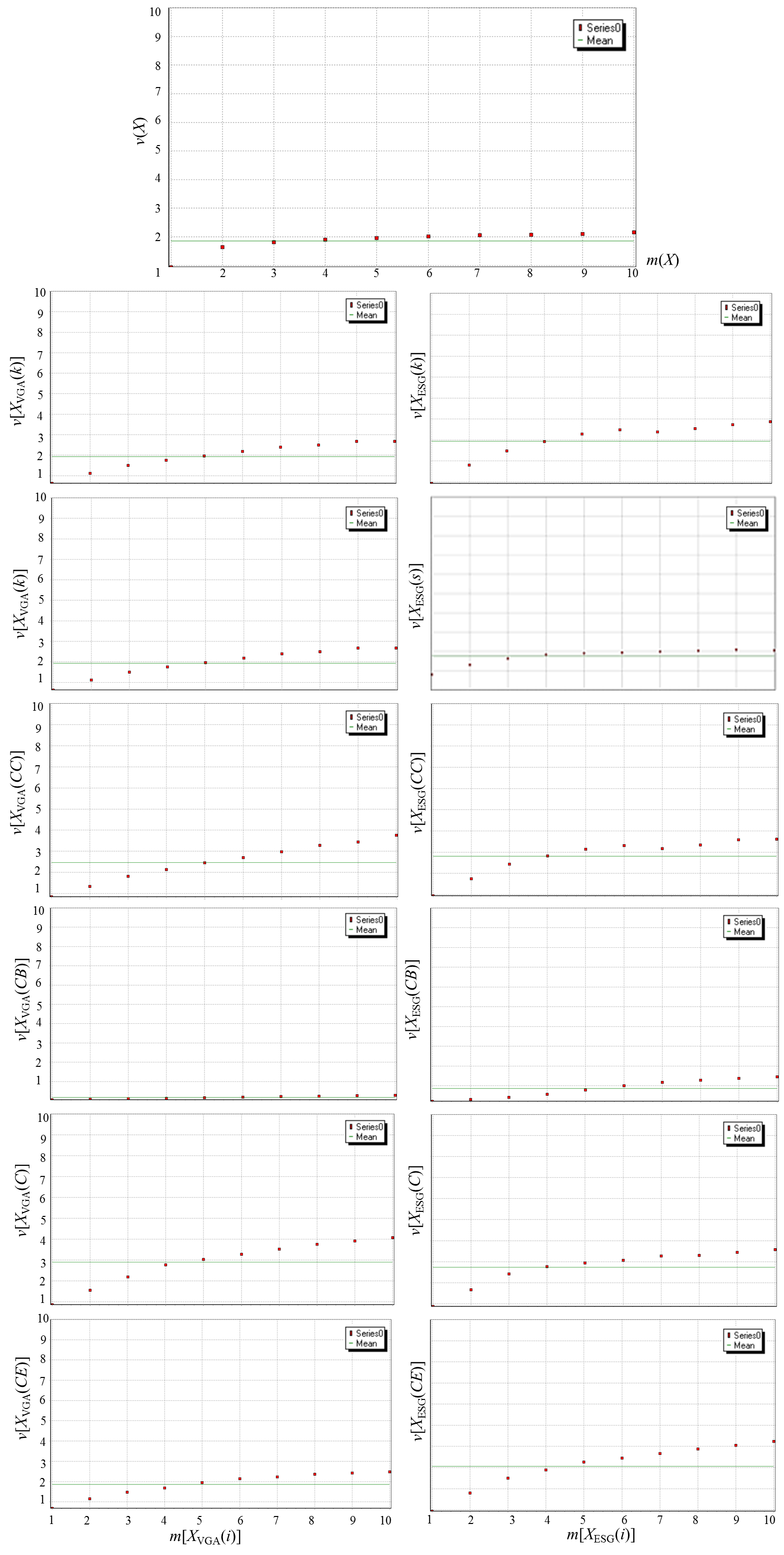
**

**Fig.S14 (part B).** Correlation (*v*) *vs*. embedding dimension (*m*) diagrams (*v*,*m*), of the VGA and ESGs node-series of strength (*s*), closeness centrality (CC), betweenness centrality (CB), clustering coefficient (C), and eigenvector centrality (CC), which are associated to the typical Lorentz chaotic time-series *X_b_*.

**The ESGA Matlab (m-file) Code**

function [ ESGA ESGA_n fc] = esga_und( x )

%ELECTROSTATIC GRAPH ALGORITHM (ESGA) this function creates an undirected graph associated to a time-series by using

%the electrostatic graph algorithm

% INPUTS

% x: a time-series vector

%

% OUTPUTS

% ESGA: the associated electrostatic graph

% ESGA_n: the complete associated electrostatic graph (i.e. prior applying the

% electrostatic threshold)

% fc: the electrostatic threshold (charge), where higher than

% (>=) fc connections are kept in the ESGA.

%

% Developed by Dimitrios K. Tsiotas, Ph.D., 27 June 2020

tic

n=length(x);

ESGA=zeros(n);

fc=sum(x)/(n-1)

for i=1:n

for j=1:n

ESGA(i,j)=(x(i)*x(j))/(i-j)^2;

end

end

ESGA_n=ESGA;

ESGA=ESGA.*(ESGA>=fc);

toc

end
